# Exosome-derived tRNA fragments tRF-GluCTC-0005 promotes pancreatic cancer liver metastasis by activating hepatic stellate cells

**DOI:** 10.1038/s41419-024-06482-3

**Published:** 2024-01-30

**Authors:** Wei Chen, Wang Peng, Ronghua Wang, Shuya Bai, Mengdie Cao, Si Xiong, Yanling Li, Yilei Yang, Jingwen Liang, Luyao Liu, Hamza O. Yazdani, Yuchong Zhao, Bin Cheng

**Affiliations:** 1 Department of Gastroenterology and Hepatology, Tongji Hospital, Tongji Medical College, Huazhong University of Science and Technology, Wuhan, 430030 China; 2 Department of Surgery, University of Pittsburgh School of Medicine, Pittsburgh, PA 15213 USA

**Keywords:** Metastasis, Cancer microenvironment

## Abstract

Early metastasis is the primary factor in the very poor prognosis of pancreatic ductal adenocarcinoma (PDAC), with liver metastasis being the most common form of distant metastasis in PDAC. To investigate the mechanism of PDAC liver metastasis, we found that PDAC cells can promote the formation of pre-metastatic niches (PMNs) through exosomes to facilitate liver metastasis in the early stage. In our study, hepatic stellate cells (HSCs) were treated with PDAC-derived exosomes (PDAC-exo), and the activation of HSCs was detected. A novel transfer RNA-derived fragment, the tRF-GluCTC-0005 was obtained by small RNA sequencing from serum exosomes of PDAC patients. Bioinformatics analysis and RNA pull-down assays revealed the interaction between WDR1 and tRF-GluCTC-0005. A KPC transgenic mouse model and an AAV-mediated sh-WDR1 mouse model were used to detect the mechanism of liver metastasis in vivo. Finally, the dual luciferase reporter assay, protein mutation truncation assay, Co-IP assay, and flow cytometry assay were used to explore the molecular mechanism in HSCs activation and PMNs formation. We found that the tRF-GluCTC-0005 in exosomes binds to the 3’ untranslated region of the mRNA of the WDRl in HSCs and increases mRNA stability. The N-terminals of WDR1 bind to the YAP protein directly, inhibit YAP phosphorylation, and promote the expression of YAP transcription factors. The tRF-GluCTC-0005 in PDAC-exo significantly recruits myeloid-derived suppressor cells (MDSCs) in the liver, creating a PMNs immunosuppressive microenvironment and further advancing liver metastasis from PDAC. Our results suggest that the key of PDAC liver metastasis is the activation of HSCs through upregulation of WDR1 by tRF-GluCTC-0005 in exosomes, which mediates the infiltration of MDSCs to form PMNs.

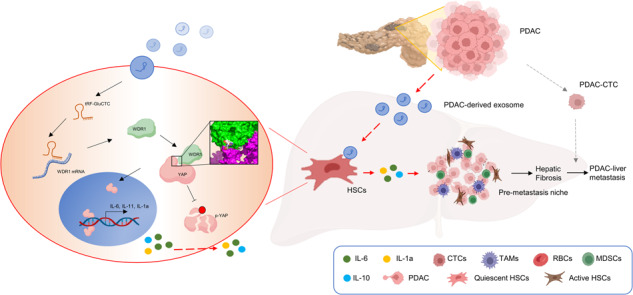

## Introduction

Pancreatic ductal adenocarcinoma (PDAC) is a highly lethal solid tumor associated with a low survival rate and high mortality. The primary treatment options for PDAC include surgical resection, chemotherapy, and radiotherapy, depending on the tumor stages [1]. Although the goal of early detection of PDAC is laudable and likely to result in significant improvement in overall survival, more than 70% of PDAC patients already lose the chance for surgical resection when diagnosed under the current modality [2]. Therefore, there is a need to develop alternative molecular diagnostic biomarkers for PDAC.

Exosomes, small vesicles approximately 30 to 200 nm in diameter with a lipid bilayer membrane structure, contain specific proteins, lipids, and nucleic acid [3–5]. These exosomes play a crucial role in intercellular communication and can facilitate various cancer-related processes, such as angiogenesis, immune modulation, and distant metastasis [3, 6–8]. Recent studies have shown that tumor-derived exosomes are involved in the formation of pre-metastatic niches (PMNs), which promote a favorable environment for the arrival and survival of metastatic cells at secondary sites [9–12]. It is crucial to PDAC’s metastasis that the PMNs shield the circulating tumor cells (CTCs) from apoptosis when they reach the metastatic location [13]. Exosomes released by cancer cells facilitate the development of PMNs by facilitating angiogenesis, stromal cell remodeling, extracellular matrix (ECM) remodeling, and oncogenic reprogramming [14–16]. Hepatic stellate cells (HSCs), which are typically dormant, become activated myofibroblasts in the presence of CTCs and tumor-derived exosomes. These activated HSCs produce cytokines that promote the infiltration of immune cells [17, 18]. The HSCs in the PMNs deposit matrix proteins, and provide a favorable fibrotic environment for tumor cell arrival [19, 20].

WD repeat-containing protein 1 (WDR1) has important roles in actin cytoskeletal remodeling that have been shown in the cytokinesis and cell migration of a variety of cells. It is engaged in actin disassembly by cutting actin filaments and dissociating actin monomers from filaments [21, 22]. It has been documented that dysregulated WDR1 controls cell invasion, migration, and proliferation in malignancies. For instance, through actin cytoskeleton-mediated control of YAP, highly expressed WDR1 stimulates the proliferation and migration of non-small-cell lung cancer cells [23]. A recent study has demonstrated that depletion of the Hippo signaling pathway led to proteasome-dependent degradation of TAZ and ultimately arrested PDAC tumor growth and liver metastasis [24].

The role of transfer RNA-derived fragments (tRFs), which are short RNAs resulting from tRNA fragmentation is of high interest in cancer research. tRFs exhibit diverse sizes and sequences and have been implicated in various aspects of cancer development and cellular stress responses [25]. According to their various locations, tRFs are categorized into five distinct types: tRF-1, tRF-2, tRF-3, and tRF-5 [26, 27]. Numerous studies have demonstrated that tRFs actively target binding RNAs or proteins to cause biological effects, including regulating protein translation, epigenetic modification, controlling cell cycle, and immune signals [25, 28–33]. Although tRFs have been deeply studied in some solid tumors, the study of tRFs in PDAC is still limited.

In this study, we aimed to investigate the diagnostic potential of tRFs in PDAC liver metastasis using RNA-sequencing techniques. We examined the expression of tRFs in peripheral blood serum exosomes obtained from 5 PDAC patients with early liver metastasis and 5 healthy volunteers. A tRF-5 type tRF-GluCTC-0005 (referred to as tRF-GluCTC) was shown to be highly increased in PDAC serum exosomes during the following qPCR validation. We observed a positive correlation between rising levels of tRF-GluCTC and the size and depth of metastatic tumors. Additionally, we found that tRF-GluCTC stimulates liver fibrosis and induces the activation of hepatic stellate cells, leading to the formation of PMNs. Mechanistically, tRF-GluCTC directly binds to WDR1 mRNA, stabilizing its expression and facilitating its interaction with YAP. This interaction facilitates the downstream molecular pathway of IL-6 and promotes PDAC liver metastasis. These findings offer potential treatment strategies, in addition to improving PDAC prognosis, surveillance, and early diagnosis of liver PMN to better control metastasis progression.

## Materials and Methods

### Patient pancreas tissue specimen and demographic information

We obtained paraffin-embedded sections of PDAC and adjacent pancreas specimens from 40 PDAC patients who underwent curative resection between 2013 and 2018 at the Tongji Hospital, Huazhong University of Science and Technology (HUST, Wuhan, China). We also obtained 73 PDAC patients’ serum samples from HUST. Clinical data associated with these specimens were recorded without patient identification. All human experiments were approved by the ethics committee of Tongji Hospital. Informed consent was obtained from all subjects. Tumor differentiation was defined according to the Edmondson grading system.

### Cell lines and cell culture

The human PDAC cell lines AsPC1, BxPC3, and Hpne were obtained from the American Type Culture Collection (ATCC, Manassas, VA, USA). Panc1, Capan2, Panc02, and 293 T cells were purchased from the Cell Resource Center, Institute of Biochemistry and Cell Biology at the Chinese Academy of Science (Shanghai, China). All cell lines were cultured in Dulbecco’s modified Eagle’s medium (DMEM; GIBCO, Grand Island, NY, USA) supplemented with 10% fetal bovine serum (FBS; GIBCO) at 37 °C and 5% CO_2_. Cells were confirmed to be free of mycoplasma using the Bimake mycoplasma detection kit.

### Exosome isolation

100 ml of cell culture supernatant from all the cell lines was collected and concentrated using a 10 kDa filter. 10 ml of the concentrate was incubated with magnetic exosome trap beads (Tymora Analytical Operations). The samples were incubated by shaking or end-over-end rotation for 60 minutes according to the manufacturer’s instructions. The supernatant was removed using a magnetic separator rack, the beads were washed once with PBS, and the exosomes were eluted by two 10 min incubations with 100 mM of fresh trimethylamine (TEA, Millipore Sigma). Simultaneously, a fraction of the concentrated supernatant was also subjected to Transmission Electron Microscopy (TEM) and western blot analysis of exosome markers.

### Transmission Electron Microscopy (TEM)

Exosomes were resuspended in 2% paraformaldehyde and loaded on carbon formvar-coated copper grids, which were subsequently stained with uranyl acetate. The exosomes were fixed overnight in 2% glutaraldehyde in 0.1 M phosphate buffer, post-fixed for 1 hour in 2% osmium tetroxide in 0.1 M phosphate buffer, dehydrated through a series of graded ethanol, and embedded in EM-bed (Electron Microscopy Sciences, Fort Washington PA). The glass coverslip was dissolved in hydrofluoric acid. 100 nm sections were cut on a Leica Ultracut EM UC7 ultramicrotome and stained with uranyl acetate and lead citrate. The grids were viewed at 80 kV in a JEOL JEM-1400 transmission electron microscope and images were captured using an AMT BioSprint 12 digital camera.

### Lentivirus production, transduction, and plasmids construction

Lentivirus-mediated tRF-GluCTC-0005 expression or silencing was achieved by the documented method [34] with some modifications. Briefly, for expressing, the synthesized tRF-GluCTC sequence was inserted into pLKD-CMV-mcherry-2A-Neo-U6-shRNA vector containing the miR-30 backbone (ObiO Technology). The plasmid and its insertion sequences were authentic by DNA sequencing. These vectors and a lentiviral vector packaging system (ObiO Technology) were then co-transfected into 293 T cells using Lipofectamine 3000. The resultant lentiviruses were designated tRF-GluCTC expression. AsPC-1 and BxPC-3 cells were infected with these lentiviruses in the presence of polybrene (Sigma-Aldrich) and selected by puromycin. The alternations of tRF-GluCTC levels in cells were detected by qRT-PCR. For constructing FLAG-tagged WDR1 expression vectors, synthesized full-length, truncated, or mutated WDR1 cDNA was respectively subcloned into the pcDNA3.1-3×FLAG vector (ObiO Technology). For constructing the HA-tagged YAP expression vector, synthesized full-length or truncated YAP cDNA was subcloned into the pcDNA3.1-HA vector (ObiO Technology).

### Transient transfection of tRF-GluCTC mimics, siRNAs, and plasmids

tRF-GluCTC mimics and inhibitors (Supplementary Table S1) were synthesized by RiboBio (Guangzhou China). cDNAs encoding WDR1 and YAP were individually subcloned into the pcDNA3.1 vector (Umine Biotechnology) and the resultant vectors were named pcDNA3.1-WDR1 and pcDNA3.1-YAP, respectively. A blank pcDNA3.1 vector served as a control. Transfection with mimics (50 nM), plasmids (1 ng/ml), or siRNAs (50 nM) was performed with Lipofectamine 3000 (Life Technologies).

### RNA isolation and reverse transcription quantitative PCR (RT-qPCR)

Total RNA extracted from cell lines and pancreatic tissue specimens with Trizol reagent (Invitrogen) was reverse transcribed with random primers or specific tRF stem-loop RT primers. Real-time quantitative PCR using SYBR Premix (DRR041A, TaKaRa, Japan) was performed as described previously [34]. U6 RNA was used as an internal control for the quantification of tRFs. A standard curve for each gene was generated from serially diluted standards, and values for unknown samples were extrapolated. All standards and samples were assayed in triplicate. The assay details and primer sequence are presented in the Supplementary section (Supplementary Table S1).

### Biotin-coupled probe RNA pull-down assay

Biotinylated WDR1 mRNA and tRF-GluCTC (RiboBio, China) pull-down assays were performed as described earlier [35]. Briefly, biotin-labeled WDR1 mRNA probes were transfected in 293 T cells in a CO2 incubator at 37 °C for 60 minutes and fixed using methanol. The cell quickly lysed with 4 ml Trizol and total RNA was extracted and sonicated. After centrifugation, 50 μL of the supernatant was used as input, and affinity streptavidin magnetic dynabeads (Invitrogen, USA) were added to the RNA fragments at 4 °C overnight. Streptavidin magnetic beads were obtained under a magnetic field. After washing with the wash buffer, the RNA complexes bound to the beads were eluted and purified with Trizol Reagent (Takara, Japan) for further analysis. The sequences are presented in the Supplementary Table S1.

### RNA FISH and immunofluorescence

Fluorescent in situ hybridization (FISH) was performed utilizing RiboBio Fluorescent In Situ Hybridization Kit (Guangzhou China). Briefly, fixed and permeabilized PDAC cells or paraffin sections were hybridized with tRF-GluCTC probes (Ribo Bio, Supplementary Table S1) overnight in a humidified chamber at 37 °C in the dark and the images were obtained with Olympus FV1000 confocal microscope (Olympus). The signals were detected using 4’,6-diamidino-2-phenylindole, and Cy3 channels. Cell nuclei were counterstained with DAPI. Antibodies against α-SMA (1:200, BOSTER) and secondary antibodies (1:500, A-21206, Invitrogen) were used for immunofluorescence staining. For immunofluorescent analysis, the tissue sections were incubated with primary antibodies followed by incubation with secondary antibodies according to the experiment design. The signals were detected by using FITC and Cy3 channels. Cell nuclei were counterstained with DAPI. Images were obtained with LSM880 confocal microscope (Zeiss). The details of the antibodies are listed in the Supplementary Table S2.

### Cell migration and invasion assay

The cell invasive and migratory ability was detected using an 8um pored, 6.5 mm polycarbonate transwell filter (Millipore, Billerica, MA, USA), according to our previous study [34]. For the migration assay, 5×10^4^ cells were seeded in the upper chamber with serum-free DMEM. DMEM medium with 10% FBS was added to the lower chamber. After incubation for 36–48 hours, the cells were fixed with paraformaldehyde and stained with a 4 g/l crystal violet solution for 0.5 hours. Cells were counted using a microscope. For the cell invasion assay, the chamber was uniformly coated with a Matrigel layer. The following procedures were the same as the migration assay. The migration and invasion experiments were performed in triplicate.

### Collagen gel lattice contraction assay

A modified in vitro cell contractility assay was carried out as studies demonstrated before [36]. Briefly, p-HSCs or LX2 cells with different treatments at a density of 2 × 10^5^ cells/ml were mixed with solubilized type I collagen (Sigma, USA) to form a cell–collagen suspension at a final concentration of 1 mg/ml. A total of 200 μl of suspension was dropped in a 24-well culture dish immediately and then incubated in a growth medium for another 3 days before the cell–collagen lattice was mechanically released from the underlying plastic substratum. Subsequently, the lattice was exposed to serum-free Dulbecco’s modified Eagle’s medium (DMEM), DMEM plus 10% fetal bovine serum (FBS), or DMEM plus 1 μM calcium ionophore (Ca-Ionophore, Sigma, USA). Serum-free DMEM was used as a negative control, while FBS was used as a positive control. The diameters before releasing the lattice and after the various exposures were recorded for relative percent contraction.

### Mice and tumor models

Six-week-old male C57BL/6 mice were obtained from Beijing Huafukang Biotechnology company and maintained in pathogen-free conditions. Animal experiments were performed according to the NIH Guide for the Care and Use of Laboratory Animals, with the approval of the Tongji Hospital Institutional Review Board. All experimental mice were gender and age-matched (ranging between 6 and 10 weeks, each group 8 animals). When grouping animals, they are randomly assigned to each experimental group to ensure that the studied variables do not result in biased data within each group. For the exosome education and splenic orthotopic injection liver metastases models, the following conditions were considered for exosome education followed by tumor challenge: Saline only, PDAC-exo, and PDAC-exo transfected with tRF-GluCTC. 40 µg of exosomes were injected into the caudal vein of naïve 6–8-week C57BL/6 mice. The injections were done every three days for 21 days. In the splenic orthotopic injection liver metastases models, 5×10^5^ luciferase-labeled Panc02 cells were injected orthotopically in the spleen of mice that received exosome injections. After 5 weeks, bioluminescence was measured 5 minutes after a tail intravenous injection administration of 100ul of potassium D-luciferin salt (30 mg/mL) dissolved in PBS (per animal). The mice were sacrificed 6 weeks post-surgery. Tumor growth was followed with a caliper, and tumor volume =XY^2^/2, X is the longest and Y is the shortest of two perpendicular diameters. The murine models of PDAC: *K-Ras*^+/LSL-G12D^; *Trp53*^LSLR172H^; Pdx1-Cre mouse model (KPC) and the *K-Ras*^+/LSLG12D^; Pdx1-Cre mouse model (KC) have been described previously [37] and were kept on a C57BL/6 background. All mice were fed ad libitum and challenged with tumors except the WT control and monitored according to IACUC guidelines and sacrificed if an excessive deterioration in health was observed.

### ^68^Ga-DOTA-FAPI PET Imaging in vivo

In vivo, small animal imaging was conducted at the Nuclear Medicine Department of Tongji Hospital, Tongji Medical College, Huazhong University of Science and Technology. Mice were fasted for 8 hours and injected with approximately 50 µCi of ^68^Ga-DOTA-FAPI via lateral tail vein (the exact dose was calculated by measuring the syringe before and after injection). Mice were maintained in cages at room temperature for 1 hour and anesthetized with isoflurane. Mice were placed on a pad in the prone position, followed by micro-PET and micro-CT imaging. ^68^Ga-DOTA-FAPI uptake was quantified by drawing region of interest (ROI) using IRIS PET/CT software and plotting maximum uptake values (SUVmax).

### Western blot analysis

Western blot analysis was performed as described previously [34]. Primary antibodies are listed in the Supplementary Table S2. Monoclonal mouse anti-GAPDH was used as an internal control.

### Immunohistochemistry (IHC)

The antibodies used for histochemical validation are listed in Supplementary Table S2. Briefly, the paraffin section was heated at 60 °C for two hours and then hydrated conventionally, following which antigen retrieval was done by steaming the slides for 20 minutes in Antigen unmasking solution Tris Based (VectorLabs Cat#H3301-250), following which slides were cooled to room temperature. Endogenous peroxidase, pseudoperoxidase, and alkaline phosphatase in FFPE sections were blocked with BLOXALL (VectorsLabs Cat # SP6000-100) for 10 minutes. Cells were then washed in IHC wash buffer (PBS with 0.1% Tween20) for 5 minutes following which they were incubated with normal goat serum (2.5%) for 20 minutes to block non-specific sites. The antibodies were then diluted in goat serum at the dilutions mentioned earlier and the sections were incubated at 4 °C overnight in a humidified chamber. The slides were then washed in wash buffer for 5 minutes and incubated for 30 minutes with ImmPRESS Universal Polymer Reagent (VectorLabs Cat# MP-7451). The slides were washed again twice with wash buffer and incubated with ImmPACT DAB EqV peroxidase substrate solution (VectorLabs Cat#SK4103-400) for 5 minutes. After repeating the wash steps twice for 5 minutes slides were then rinsed in tap water. The slides were counterstained with Hematoxylin QS counterstain (VectorLabs Cat# H3404-100) for 60 seconds and rinsed in tap water. The slides were dehydrated conventionally and then mounted with Vectamount permanent mounting medium (VectorLabs Cat# H5000-60). The blocking and antibody incubations were done in 2.5% horse serum according to the manufacturer’s instructions. Assessment of IHC staining scores was independently performed by two pathologists who were blinded to the clinical data. The intensity of staining was scored on a scale as negative (0, no staining), weak (1, light yellow), moderate (2, brown), or strong (3, brown red). The extent of the staining was evaluated according to the percentage of positive areas of cells in relation to the whole area, which was scored on a scale of 0–4, 0 (0), 1 (1–25%), 2 (26–50%), 3 (51–75%), and 4 (76–100%). Protein expression levels (range 0–12) were calculated by multiplying the staining intensity and positive staining score. Then, we divided the patients into two groups (grade < 6, low expression; grade >= 6, high expression) and performed survival analysis.

### Luciferase reporter assay

293 T cells were seeded in 24-well plates (3 × 10^4^ cells/well). Negative control mimics or tRF-GluCTC mimics were co-transfected with the reporter plasmid into 293 T cells using Lipofectamine 3000. After 48 h, the cells were collected and lysed with passive lysis buffer. The luciferase signal was detected using the luciferase reporter assay kit (Promega, Madison, Wisconsin, USA) and measured using BMG FLUOstar OPTIMA Microplate Reader (BMG LABTECH, Cary, North Carolina, USA). Firefly luciferase activity was normalized to Renilla luciferase activity.

### Flow cytometry analysis

Antibodies specific are listed in Supplementary Table S2. The flow cytometry data were acquired using BD LSRII flow cytometer and analyzed by FlowJo Software (Tree Star). Lytic histiocyte cells (2.5 * 10^6^ cells) were centrifuged. The cell pellets were suspended in FACS buffer (PBS containing 0.5% fetal bovine serum) and then labeled with antibodies at 4 °C for 30 minutes. Cells were washed twice, re-suspended in FACS buffer, and analyzed with the FACS Calibur machine using CellQuest software (BD Biosciences).

### multiplex and ELISA analysis

Human IL-6, IL-10, Mouse IL-1a concentration was measured in exosome treatment and normal control HSCs CMs using the ELISA kits (Servivrbio, GEH0003–96T, GEH0001-96T, GEM0036-96T). Human IL-6, IL-10, and mouse IL-1a concentrations were simultaneously measured in exosome treatment and normal control HSCs CM using a bio-Plex ProTM cytokine Multiplex Immunoassay, according to the manufacturer protocol. Data were analyzed using a bio-plex 200 instrument and bio-Plex software.

### Gene Set Enrichment Analysis (GSEA) analysis

The gene expression profiles and clinical data of PDAC patients were obtained from the TCGA data portal (https://www.cancer.gov/tcga/) in November 2021. We analyzed HSCs in Panc02 and KPCY pancreatic cancer liver metastasis model, respectively. The median value was set as the cutoff. Next, GSEA v4.0.1 was applied to perform GO analysis and KEGG analysis to investigate the functions of HSCs in liver metastases from pancreatic cancer.

### Analysis of GEO bulk RNAseq datasets

Human-derived expression microarray data (GSE71729), and mouse-derived RNA-seq data (GSE148139, GSE160541) for pancreatic cancer were obtained from the Gene Expression Omnibus (GEO) database. Differential expression analysis was performed for the following comparisons: Mouse HSC-derived CAF from Panc02 liver metastasis model *vs*. quiescent mouse HSCs, Mouse HSC-derived CAF from KPCY liver metastasis model *vs*. quiescent mouse HSCs in GSE160541 (log2FoldChange > 2); Mouse HSCs treated by mouse PDAC cell lines (Panc02, and KPC) derived exosomes *vs*. Mouse HSCs treated by mouse normal pancreatic duct epithelial cells derived exosomes in GSE148139 (log2FoldChange > 0.5); and 61 metastatic PDAC tumors *vs*. 46 normal pancreas tissues in GSE71729 (log2FoldChange > 0.15). The up-regulated genes from all comparisons were overlapped to define a 25-gene signature of HSCs activation by PDAC-exosomes.

### single-cell RNA-seq analysis

Public single-cell RNA-seq data of normal liver specimens (SCP2154) and PDAC-metastatic liver specimens (GSE156405, GSE205013, and SCP1644) were extracted from datasets obtained from the Gene Expression Omnibus (GEO) database and the Broad Institute Single Cell Portal. Dimension reduction and cell clustering followed the recommendations of the Seurat package. The cells were annotated according to cell markers as B cells (MS4A1, CD79A), DC cells (LILRA4, CLEC4C), endothelial cells (VWF, CDH5, CLDN5, PLVAP), liver epithelial cells (ALB, APOB), pancreatic epithelial cells (KRT19, SLPI, MUC1), HSCs (ACTA2, COL1A1, PDGFRB, BGN), myeloid cells (AIF1, LYZ, CSF1R, CD14), NK/T cells (CD3D, CD3E, GZMA, TRAC) and unclassified cells. The tSNE plots were used to show the annotated cell types and normalized expression levels of indicated genes.

### Mass spectrum analysis

Eluate-containing proteins associated with WDR1 from protein immunoprecipitation were firstly digested into peptides and then subjected to mass spectrum analysis with TripleTOF® 6600 mass spectrometer and Eksigent NanoLC 2D Plus system (AB SCIEX). ProteinPilot 5.0 software (AB SCIEX) was used for analysis and proteins were ranked by the exponentially modified protein abundance index (emPAI) (Supplementary Table S3).

### In silico molecular docking analysis

Using the PyMOL Molecular Graphics System, vers.1.2r3pre (Schrödinger, LLC, Palo Alto, CA2002, USA), the structure was transformed into the protein databank (PDB). The 3D structure of the receptors and crystal structures of YAP (PDB; 1SSE) was retrieved from the PDB. In this study, InstaDock was used for molecular docking-based virtual screening. We performed blind docking in which the entire receptor protein structure was used to find the best binding pocket by the compounds. The output files of InstaDock were in log-files and out-files, which were extracted based on the proteins’ binding affinity and docking conformations towards WDR1 used for further analyses. The removal of water molecules, the addition of hydrogen atoms, and Kolmman charges in the receptor were made as prerequisites of pre-docking. Molecular docking studies were conducted using AutoDock VINA software as described in our previous studies [38, 39]. The best poses of ligand-receptor complexes of hydrogen bonds and electrostatic and hydrophobic interactions were expressed as binding energy values (kcal/mol) to represent docking results. To visualize H-bond interactions, binding affinities, interacting amino acid residues, atoms binding to the ligands and receptors, and 3D graphical representations of ligand-receptor complexes were made using PyMOL software.

### Statistical methods

Data was presented as mean ± SD in each experiment. Statistical significance was considered as *P* < 0.05 in each experiment. Statistical analysis was processed using R (v4.0.2) and GraphPad Prism 9.0 software. We chose appropriate statistical tests according to the types of data. For the data approximately normally distributed, student t-test and one-way ANOVA were performed. Wilcoxon signed-rank test was applied to analyze the nonnormal distribution data. Survival analysis was evaluated using a log-rank test. *P* < 0.05 was regarded as statistically significant.

## Results

### HSCs are activated in hepatic metastases of PDAC

**Fig. 1:**
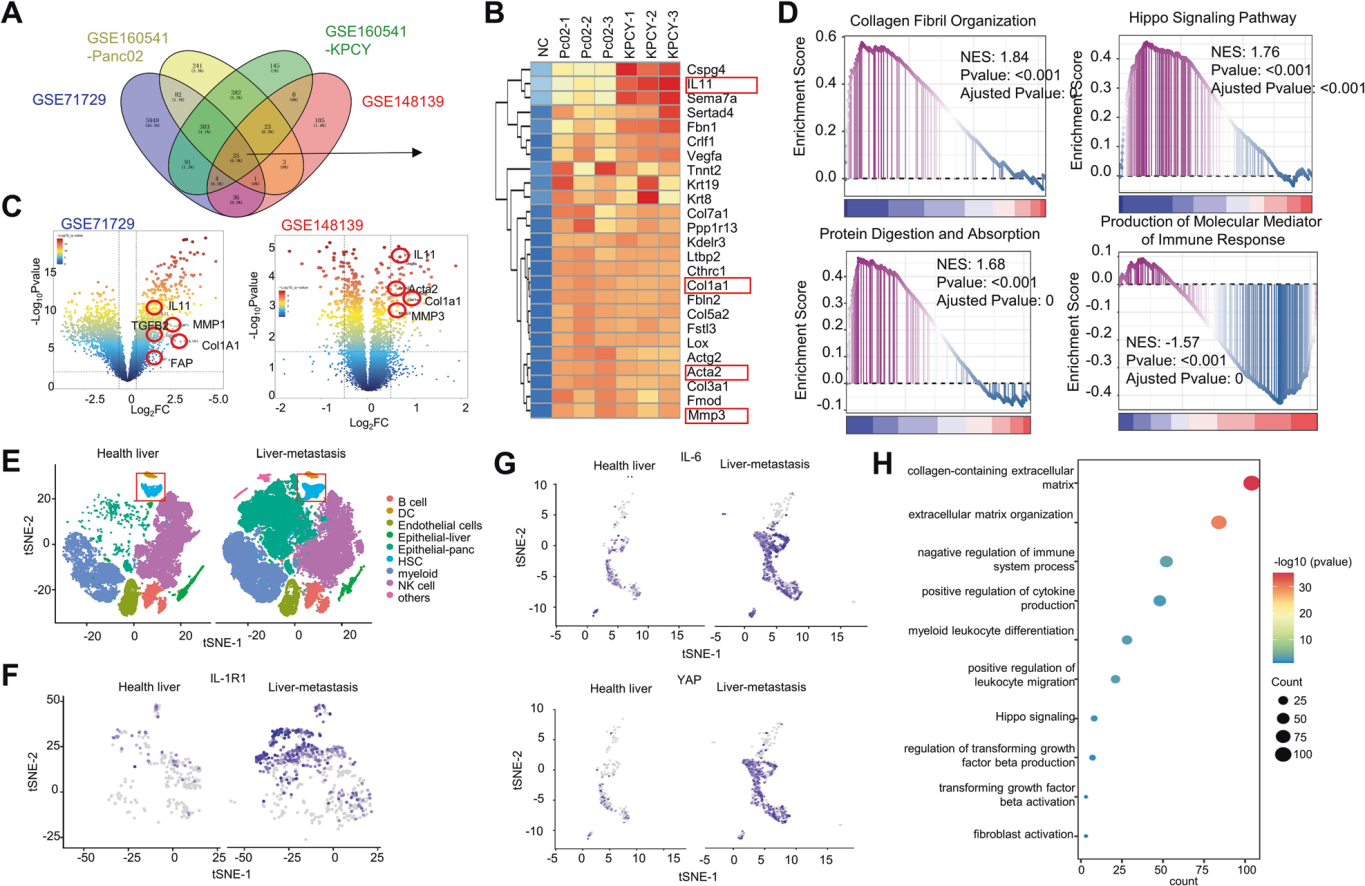
HSCs are activated in hepatic metastases of PDAC. **A** Venn diagram of differentially expressed genes derived from GEO databases (GSE160541, GSE71729, and GSE148139). The numbers in each circle (intersection) represent the unique number of differentially regulated genes in respective comparisons; 25 genes were expressed commonly in three different databases and were used for further analysis. **B** The heat map shows the expression of 25 common overexpression genes. **C** Volcano plot of selected differentially regulated genes related to HSCs activation protein and extracellular matrix proteins in PDAC-derived exosome treated group vs. HSCs untreated group. The vertical dashed line indicates an adjusted *P* < 0.05, and the horizontal dashed lines indicate an absolute log2 FC > 2. **D** GSEA analysis demonstrated that highly expressed genes in HSCs were predominantly enriched in protein digestion and absorption, collagen fibril organization, and Hippo signaling pathways. **E** Concatenated Uniform Manifold Approximation and Projection (UMAP) plot shows annotated clusters from 90,583 single cells undergoing RNA sequencing from PDAC liver metastasis foci tissue and healthy liver tissue. **F** Superimposed UMAP plot highlights IL-1r1 positive HSCs subclusters in healthy liver tissue and liver metastasis tissue. **G** Superimposed UMAP plot highlights IL-6 and YAP positive HSCs subclusters in healthy liver tissue and liver metastasis tissue. **H** A bubble plot of pathway enrichment analysis highlights the strongly differentially enriched pathways in the HSCs cluster using Kyoto Encyclopedia of Genes and Genomes with adjusted *P* < 0.05 was selected.

To investigate the effects of primary PDAC on the activation of HSCs, sequencing data from human-derived (GSE71729) and mouse-derived (GSE148139, GSE160541) pancreatic cancer released mediators were obtained from the public database Gene Expression Omnibus (GEO) datasets (Fig. 1A). In GSE71729, 145 primary and 61 metastatic PDAC patients tumors were selected in our study. In GSE148139, an examination of mice primary HSCs RNA sequencing comparing the PDAC-exosomes treatment group with the control group was selected. And in GSE160541, bulk RNA sequencing was performed in 3 isolates of quiescent mouse HSCs; 3 isolates of HSC-derived CAF from Panc02; and 3 isolates of HSC-derived CAF from KPCY. Among these datasets, 25 genes were significantly upregulated (Fig. 1B; log[FoldChange] > 2, *p* < 0.05). Most of these genes were associated with hepatic fibrosis and cytokine secretion in HSCs (Fig. 1C). The highly expressed genes Col1a1, Acta2, and MMP3, which are related to HSCs activation, were identified from the GSE148139 and GSE71729 databases. Furthermore, gene-set enrichment analysis (GSEA) demonstrated that highly expressed genes in HSCs were predominantly enriched in protein digestion and absorption, collagen fibril organization, and Hippo signaling pathways induced by mediators secreted by PDAC cells. Additionally, the production of molecular mediators of the immune response was downregulated (Fig. 1D).

We then conducted single-cell sequencing analysis on PDAC liver metastases and normal liver tissue to investigate the presence of HSCs. We analyzed a total of 90,583 cells after filtering low-quality cells and visualized them using the Uniform Manifold Approximation and Projection (UMAP) plot. These cells were integrated well by removing the batch effect and clustered into 9 clusters (Fig. 1E). We defined the cell types of each cluster according to their signature genes and canonical cell-type markers curated from literature (Supplementary Fig. 1A). Our analysis revealed higher expression levels of IL1r1 in HSCs within the liver metastasis group compared to the normal group (Fig. 1F). In addition, we also detected the expression of IL-6 and YAP genes targeting a subpopulation of HSCs. These results fully corroborate the activation of HSCs in PDAC liver metastasis PMN (Fig. 1G). Enrichment analysis of single-cell sequencing data for PDAC liver metastases showed that HSCs-specific differential genes were enriched in collagen production, formation of an immunosuppressive environment, cytokine secretion, and activation of the Hippo signaling pathway (Fig. 1H). These results demonstrate that PDAC cells can promote hepatic fibrosis and activate HSCs.

### PDAC-derived exosomes activate hepatic stellate cells

Since exosomes play an important role in cell and organ communication [40], we hypothesize that PDAC-derived exosomes could activate HSCs during liver metastasis of pancreatic cancer. We next extracted exosomes of PDAC cell lines and subjected them to quality identification. Transmission electron microscopy (TEM) observation and exosome nanoparticle tracking analysis (NTA; Supplementary Fig. 1B, C) confirmed that the extracted exosomes had a diameter of approximately 80 nm and intact membranes. Exosomal signature proteins (CD9, CD81, CD63) were also detected to prove the successful extraction of the exosome (Supplementary Fig. 1D).

**Fig. 2:**
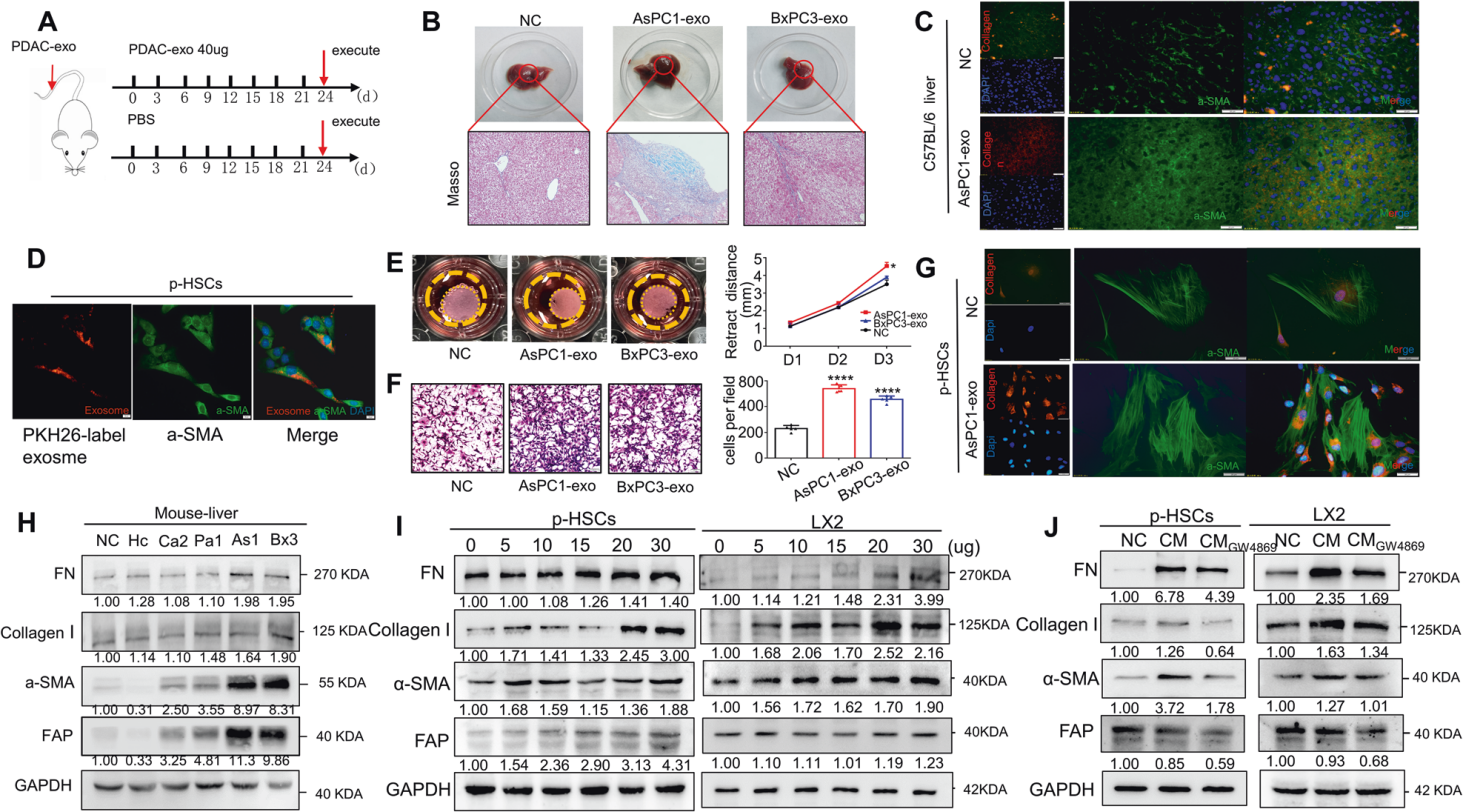
PDAC-derived exosomes activate hepatic stellate cells. **A** Schematic diagram of diverse PDAC cell lines-derived exosome promoting liver fibrosis in mice. 40ug PDAC-exo was treated in mice every 3 days and all the mice were executed after 3 weeks. **B** The degree of liver fibrosis was shown in Masson staining. **C** The fluorescence intensity of α-SMA (green) and collagen I (red) was increased in the AsPC1-derived exosomes stimulated group by immunofluorescence analysis. **D** Representative images of interaction between PDAC-derived exosomes and patient-derived primary hepatic stellate cells (p-HSCs). Immunofluorescence analysis of p-HSCs after stimulation by PKH26-labeled exosomes (red). Scale bar, 20 μm. **E** Collagen gel containing p-HSCs was treated with AsPC1-Exo or BxPC3-Exo 10ug per well or without treatment (NC). Statistical significance was relative to the NC group. **F** Transwell migration assays showed the migration abilities in p-HSCs stimulated by AsPC1 and BxPC3 groups. Scale bar, 100 μm. **G** Representative images of immunofluorescence analysis. The fluorescence intensity of α-SMA (green) and collagen I (red) was increased after the ASPC1-derived exosome stimulation. Scale bar, 20 μm. **H** Immunoblot showing the extent of hepatic fibrosis in mice stimulated with exosomes from different cell lines of PDCA by tail vein injection. **I** p-HSCs and LX2 cells were treated with or without different concentrations of exosomes, and harvested at the same times, protein levels of Fibronectin, α-SMA, collagen I and FAP were analyzed by immunoblotting. **J** Effect of GW4869 (a N-SMase inhibitor, an inhibitor of exosome release) on activation effect of ASPC1 coculture with LX2 cells or the p-HSCs. Each experiment was performed three times independently and the results are presented as mean ± SD. Student’s t-test was used to analyze the data; *p < 0.05, ****p < 0.0001.

To investigate the specific effects of PDAC-derived exosomes on liver in vivo, PDAC-exo was injected into C57BL/6 mice via the tail vein. As shown in Fig. 2A, mice were treated with 40 μg of PDAC-exo every 3 days for 3 weeks, after which the degree of liver fibrosis was assessed, and the expression of α-SMA and Collagen I in the liver was detected. The results showed PDAC-exo-treated mice exhibited severe liver fibrosis (Fig. 2B, C), and increased expression of extracellular matrix protein (Fig. 2H).

The activation of HSCs is an important cause of liver fibrosis. To investigate whether HSCs are activated by uptake exosomes, we isolated primary hepatic stellate cells (p-HSCs) from surgical specimens of patients’ livers in Tongji Hospital. PKH26-labeled exosomes were added to a culture system containing p-HSCs. After 12 hours, red fluorescence was observed in p-HSCs, indicating the uptake of exosomes by HSCs (Fig. 2D). The collagen gel contraction assay and transwell migration assay were performed. As expected, AsPC1- and BxPC3-derived exosomes had the greatest impact on the contractility and migration ability of p-HSCs (Fig. 2E, F, Supplementary Fig. 1E, F). Under fluorescence microscopy, PDAC-exo-treated p-HSCs showed the increasing fluorescence intensity of α-SMA, along with significant increases in cell volume and synapses (Fig. 2G). Increasing the level of PDAC-exo in p-HSCs and LX2 cells resulted in a significant upregulation of extracellular matrix proteins (fibronectin and collagen I) and HSC activation proteins (α-SMA and FAP) (Fig. 2I, Supplementary Fig. 1G). To further investigate the role of exosomes, PDAC cells were treated with the exosome-release inhibitor GW4869, and the supernatant was added to p-HSCs and LX2 respectively. Inhibition of HSC activation proteins and extracellular matrix protein expression was observed in both p-HSCs and LX2 cells (Fig. 2J, Supplementary Fig. 1H). Therefore, these findings confirm that PDAC-exo promotes hepatic stellate cell activation and induces liver fibrosis.

### The role of tRF-GluCTC in predicting liver metastasis in PDAC patients

**Fig. 3:**
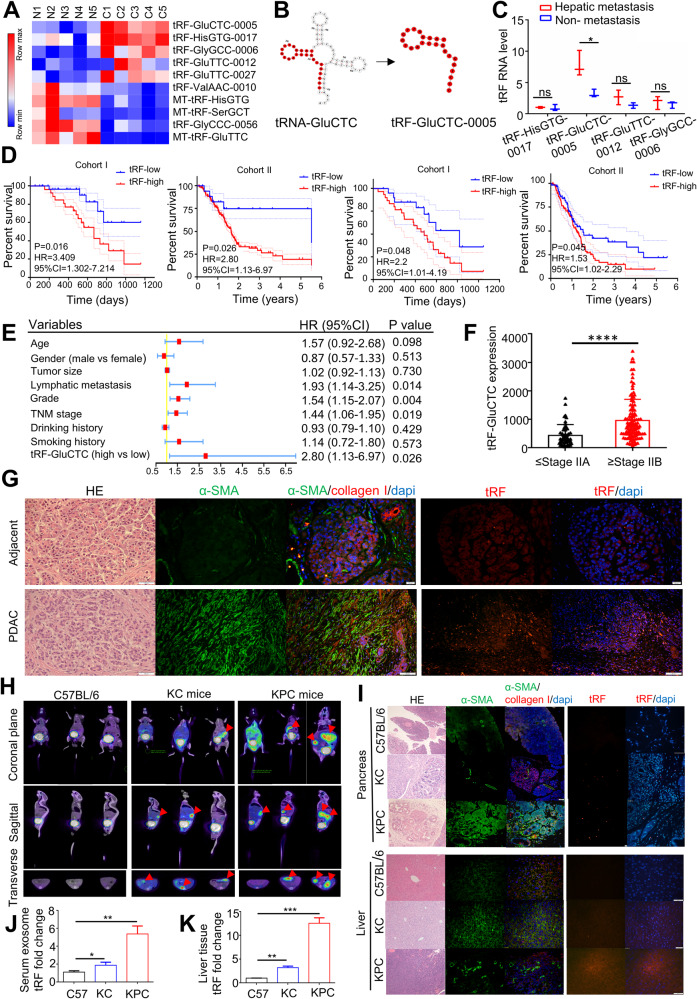
The role of tRF-GluCTC in predicting liver metastasis in PDAC patients. **A** The heat map presentation showed the top 10 tRFs small RNA sequencing from 5 PDAC with liver metastatic patients (C1-5) and 5 healthy volunteers (N1-5) peripheral blood supernatants. **B** Schematic diagram of the shearing of tRF-GluCTC-0005 from the mature tRNA-GluCTC. **C** Expression of different tRFs in serum exosomes from PDAC patients with or without liver metastases. **D** Kaplan–Meier curves for OS (left) and DFS (right), high tRF-GluCTC expression versus tRF-GluCTC expression for cohort I (*n* = 73) and cohort II (*n* = 151). **E** Forest plots of univariate and multivariate analysis for risk factors associated with OS and DFS. **F** tRF-GluCTC levels were significantly lower in stage I/IIA tumors than in stage IIB/III/IV tumors, by Wilcoxon rank-sum test. *n* = 45 stage I/IIA tumors and *n* = 106 stage IIB/III/IV tumors for cohort II. **G** Representative images of immunofluorescence analysis. The fluorescence intensity of α-SMA (green) and collagen I (red) was higher in PDAC tissues than in adjacent tissues. Scale bar, 20 μm. The expression of tRF-GluCTC in PDAC tumor tissues and adjacent tissues was analyzed by FISH. **H** Exemplary images of three different planes (coronal plane, sagittal plane, and transverse plane) of PDAC tumor, ^68^Ga-DOTA-FAPI uptake 60 min after application. **I** Representative images of liver tissue and pancreas tissue in KPC mice and C57BL/6 mice by Immunofluorescence analysis. The expression of tRF-GluCTC in the PDAC liver-metastasis tumor tissues of KPC mice and normal liver tissues of C57BL/6 was analyzed by FISH. **J** The expression of tRF-GluCTC in the serum exosomes of KC mice, KPC mice, and c57bl/6 mice and K in liver tissues of those mice. Each experiment was performed three times independently and the results are presented as mean ± SD. Wilcoxon rank-sum test and Student’s t-test were used to analyze the data; ns = no sense, *p < 0.05, ****p < 0.0001.

So far we have shown that PDAC-derived exosomes are the potential carrier and initiator of liver fibrosis. To further explore PDAC-exo contain tRFs in their cargo that may play a role in HSC activation, we collected peripheral blood supernatants from 5 PDAC patients with early metastasis (C1-5) and 5 healthy volunteers (N1-5) in Tongji Hospital. After extracting exosomes, we performed small RNA sequencing to identify differentially expressed tRFs. From the initial analysis of 431 tRFs, we selected the top 10 genes with the most significant differences for heat map presentation (Fig. 3A). Meanwhile, we examined the expression of tRFs in a variety of cancer tissues in the online dataset tdRFun. Remarkably, tRF-GluCTC-0005 was found to be significantly overexpressed in PDAC tissues (Suppl. Fig. 2A).

tRF-GluCTC-0005 (hereafter referred to as tRF-GluCTC) is a cleavage fragment of tRNA-GluCTC, 24 bases in length, formed by Dicer enzyme shearing from the 5’ end of tRNA, and contains the D-loop of the dihydrouridine region (Fig. 3B). We isolated exosome from the peripheral blood samples of 73 PDAC patients (cohort I, *n* = 73) collected at Tongji Hospital. We combined tRF-GluCTC with the traditional diagnostic marker CA19-9 to evaluate its diagnostic efficiency. The expression of tRF-GluCTC in peripheral blood serum exosomes showed higher sensitivity and specificity compared to CA19-9 (Supplementary Fig. 2B).

To validate the specificity of tRF-GluCTC as a potential biomarker for liver metastasis, we compared its expression in PDAC patients with or without liver metastases. Using RT-qPCR on peripheral blood exosomes, we detected the expression of 4 candidate tRFs in 5 PDAC patients with liver metastases and 5 PDAC patients without liver metastases. Notably, tRF-GluCTC displayed significant expression differences, indicating its potential association with liver metastasis (Fig. 3C). We then examined the expression of tRF-GluCTC and the control tRF in the serum and tissue of PDAC patients in cohort I and cohort II. The results demonstrated significantly higher expression of tRF-GluCTC in serum exosomes of patients with liver metastases compared to those without liver metastases in cohort I (OR = 4.125, *p* = 0.0292, Suppl. Fig. 2C). Similarly, patients with high tissue expression of tRF-GluCTC had a greater likelihood of liver metastasis in cohort II (OR = 2.982, *p* = 0.0334, Supplementary Fig. 2D).

To assess the prognostic value of tRF-GluCTC, we performed a Kaplan-Meier survival analysis. Patients with high tRF-GluCTC levels exhibited significantly shorter overall survival (OS) and disease-free survival (DFS) compared with low tRF-GluCTC levels in both cohort I (*n* = 73) and cohort II (*n* = 151, Fig. 3D). Furthermore, multivariate analyses using Cox proportional hazards regression demonstrated that tRF-GluCTC expression served as an independent prognostic factor for overall survival in PDAC patients (*p* = 0.026, Fig. 3E & Table 1). We then compared tRF-GluCTC levels in tissues according to different tumor stages in cohort I. The results revealed that tRF-GluCTC levels were significantly higher in advanced-stage tumors (stages IIB/III/IV) than in early-stage tumors (stages I/IIA; Fig. 3F).

**Table 1:** Risk factors related to overall survival were identified by univariate and multivariate Cox regression analysis in cohort I (n = 73) and cohort II (n = 151).

	Cohort1 (*n* = 73)		Cohort2 (*n* = 151)		Pooled samples (*n* = 224)	
	hepatic metastasis (*n* = 21)	Non- metastasis (*n* = 52)	hepatic metastasis (n = 33)	Non- metastasis (n = 118)	hepatic metastasis (*n* = 54)	Non- metastasis (*n* = 170)
Age, Mean (SEM)	57.095 (8.24)	60.711 (9.99)	61.515 (11.64)	66.423 (9.99)	60.245 (9.81)	62.15 (12.61)
Sex, *n* (%)						
Male	13 (61.9)	37 (71.2)	16 (48.5)	64 (54.2)	29 (53.7)	101 (59.4)
Female	8 (38.1)	15 (28.8)	17 (51.5)	54 (45.8)	25 (46.3)	69 (40.6)
Differentiation, *n* (%)						
Well	1 (5)	6 (11.5)	2 (6.1)	9 (7.6)	3 (5.6)	15 (8.8)
Moderate	3 (14)	22 (42.3)	4 (12.1)	21 (17.8)	7 (13.0)	43 (25.3)
Poor	10 (48)	14 (26.9)	27 (81.8)	79 (66.9)	37 (68.5)	93 (54.7)
Unclear	7 (33)	10 (19.2)	0	9 (7.6)	7 (13.0)	19 (11.2)
Lymph node metastasis, *n* (%)						
Positive	18 (85.7)	35 (67.3)	30 (90.1)	74 (62.7)	48 (88.9)	109 (64.1)
Negative	0	7 (13.4)	0	43 (36.4)	0	50 (29.4)
Unclear	3 (14.3)	10 (19.2)	3 (9.0)	1 (1.0)	6 (11.1)	11 (6.5)
Vascular invasion, *n* (%)						
Yes	15 (71.4)	32 (61.5)	33 (100)	110 (93.2)	48 (88.9)	142 (83.5)
No	2 (9.5)	15 (28.8)	0	1 (1.0)	2 (3.7)	16 (9.4)
Unclear	4 (19.0)	5 (9.6)	0	7 (5.9)	4 (7.4)	12 (7.1)
TNM stage, *n* (%)						
I	0	8 (15.4)	0	9 (7.6)	0	17 (10.0)
II	0	15 (28.8)	0	101 (85.6)	0	116 (68.2)
III	0	17 (32.7)	0	4 (3.4)	0	21 (12.4)
IV	21 (100)	12 (23.1)	33 (100)	4 (3.4)	54 (100)	16 (9.4)
Treatment, *n* (%)						
Chemotherapy only	17 (81.0)	33 (63.5)	9 (3.0)	18 (15.3)	26 (48.1)	51 (30.0)
Surgery only	0	0	20 (60.6)	68 (57.6)	20 (37.0)	68 (40.0)
Surgery + chemotherapy	4 (19.0)	19 (36.5)	4 (12.1)	32 (27.1)	8 (14.8)	51 (30.0)

*SEM* standard error of mean.

To further confirm the expression of tRF-GluCTC in pancreatic cancer tissues, we obtained surgical specimens from PDAC patients. Immunofluorescence staining revealed increased fibrotic features and higher fluorescence intensity of alpha-smooth muscle actin (α-SMA) and collagen I in pancreatic cancer tissues compared to paracancerous tissues. Additionally, fluorescence in situ hybridization (FISH) staining demonstrated significantly higher fluorescence intensity of tRF-GluCTC in pancreatic cancer tissues compared to paracancerous tissues (Fig. 3G).

To further investigate the changes in the expression of tRF-GluCTC during different stages of PDAC development, including acinar-to-ductal metaplasia (ADM) and mouse pancreatic intraepithelial neoplasia (mPanIN), we used the Kras-mediated mouse model of spontaneous pancreatic cancer in LSL-Kras^G12D/+^; Pdx1-Cre (KC) mice, as well as LSLKras^G12D/+^; Trp53^fl/+^; Pdx1-Cre (KPC) mice (Supplementary Fig. 2E). First, we employed positron emission tomography/computed tomography (PET/CT) imaging with ^68^Ga-DOTA-FAPI on KC, KPC, and wild type (WT) mice. The ^68^Ga-DOTA-FAPI Micro-PET data revealed that the KPC mice developed pancreatic cancer with significant liver metastases, whereas KC mice exhibited a slower progression of pancreatic cancer (The red arrows represent pancreatic cancer foci. Fig. 3H). Histological examination through hematoxylin and eosin (H&E) staining demonstrated that PDAC cells in KPC mice had infiltrated the basal lamina and formed diffuse tumors in the pancreas. Conversely, KC mice showed no penetration of the basal lamina, forming ADM or mPanIN (Fig. 3I).

Furthermore, the expression of α-SMA was significantly increased in liver issues of KPC mice compared with KC mice and WT mice. The FISH analysis revealed that the fluorescence intensity of tRF-GluCTC was highest in KPC mice compared to KC and WT mice (Fig. 3I). Additionally, we extracted peripheral blood serum exosomes from KPC, KC, and WT mice, and the RT-qPCR results showed that tRF-GluCTC expression was highest in the peripheral blood exosomes of KPC mice (Fig. 3J). Similarly, the expression of tRF-GluCTC was highest in the liver tissue of KPC mice (Fig. 3K). Taken together, these findings demonstrated that the expression of tRF-GluCTC in peripheral blood exosomes and liver tissues increased significantly with the progression of PDAC.

### Exosome-derived tRF-GluCTC promotes PDAC liver metastasis by activating HSCs

To investigate the specific molecular mechanisms of tRF-GluCTC in PDAC liver metastasis, firstly, we transfected mimics and inhibitors of tRF-GluCTC into AsPC1 cells and assessed the invasion and migration abilities of AsPC1 cells (Supplementary Fig. 3A). Next, we extracted exosomes from the supernatants of AsPC1 cells transfected with mimic and inhibitor. We observed that only the expression of tRF-GluCTC was altered after exosome treatment in p-HSCs, while the expression of other tRFs remained unaffected (Supplementary Fig. 3B).

**Fig. 4:**
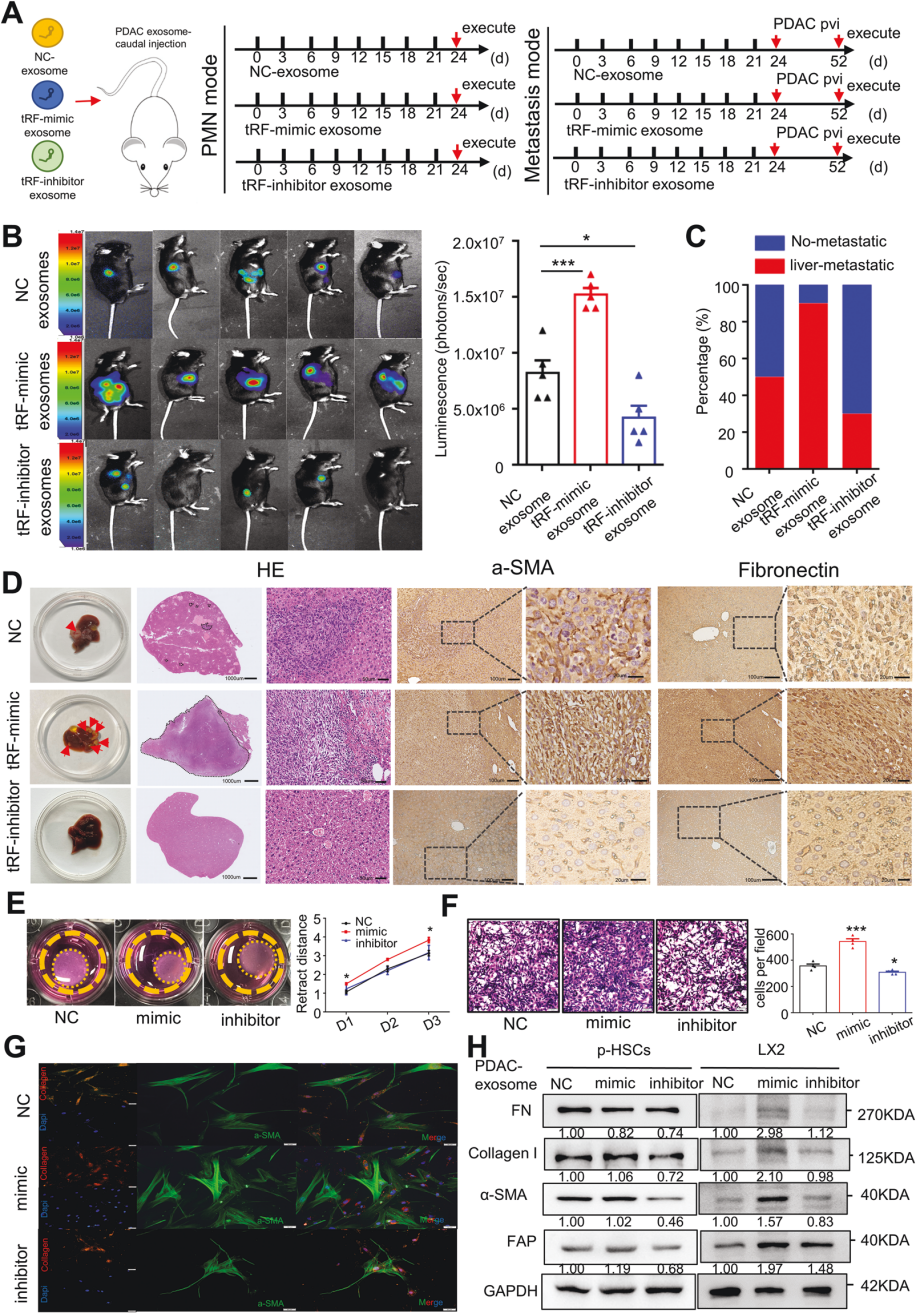
Exosome-derived tRF-GluCTC promotes PDAC liver metastasis by activating HSCs. **A** Schematic diagram of two different animal models to explore the function of tRF-GluCTC. The first animal model was injected 40ug of NC-exo, tRF-mimic-exo or tRF-inhibitor-exo through the tail vein of mice every three days for three weeks (left). The second animal model is after three weeks of PDAC-exo injected in the mice, 2*10^6^ Panc02 cells were injected into the mouse spleens immediately (right). PVI: Portal vein injection. **B** IVIS imaging was applied to determine the PDAC liver-metastasis foci in situ. The representative images were acquired with the same exposure. Bioluminescence intensity of the exosomes transfected NC, mimic, and inhibitor group at the same time point. **C** Tumor metastasis in mice from each group. **D** Representative images of PDAC-liver metastasis tissue by IHC. Scale bar, 20 μm (left), 50 μm (right). **E** Collagen gel containing p-HSCs was treated with 10ug/well exosome transfected with the tRF-GluCTC mimic and inhibitor or without treatment (NC). Representative collagen gel images from 3 independent experiments are shown. **F** Transwell migration assays showed the migration abilities in p-HSCs stimulated by tRF-exosome transfected mimic or inhibitor. Scale bar, 50 μm. **G** Representative images of p-HSCs in those groups by immunofluorescence. Scale bar, 50 μm. **H** Western blot analysis of the expression of fibronectin and α-SMA in LX2 cells and p-HSCs after stimulated by the exosome transfected the tRF-GluCTC mimic and inhibitor. Each experiment was performed three times independently and the results are presented as mean ± SD. Student’s t-test was used to analyze the data; *p < 0.05, ***p < 0.001.

To better understand the role of PDAC-exo-induced liver PMNs and metastasis, we established 2 mouse models in vivo (Fig. 4A). The first animal model was aimed to establish the PMNs of PDAC. We injected 40ug of Ctrl-exo, tRF-mimic-exo, or tRF-inhibitor-exo through the tail vein of mice every three days for a total of three weeks, a process referred to as “education”. We obtained mouse livers and detected the expression of HSCs-activated proteins and extracellular matrix proteins in mouse livers using immunohistochemical staining. We observed a significant increase in liver fibrosis in tRF-mimic-exo stimulated mice, whereas liver fibrosis in tRF-inhibitor exosome-stimulated mice was not significant (Supplementary Fig. 4A). Western blot analysis of mouse liver proteins confirmed that the expression of α-SMA and FAP was higher in the tRF-mimic group, and similarly the expression of extracellular matrix proteins was also increased (Supplementary Fig.  4B).

The second animal model focused on liver metastasis of pancreatic cancer. Following three weeks of PDAC-exosome “education” in the mice, 2*10^6^ Panc02 cells were injected into the portal vein. The IVIS Lumina imaging system was used to monitor the liver metastasis weekly. In the fourth week, we found the fluorescence intensity in mouse livers was higher in the tRF-mimic-exo education group, compared with the Ctrl-Exo education group and tRF-inhibitor-exo education group (Fig. 4B). We quantified the proportion of liver metastases that occurred in the different groups, and the results demonstrated that the tRF-GluCTC mimic group had a higher propensity for liver metastasis compared to the control group (Fig. 4C). Subsequently, mice were sacrificed, and their livers were harvested for IHC staining. The degree of fibrosis and rates of metastases were significantly higher in mice with the tRF-mimic-exo education group compared to those in the Ctrl-exo education group (Fig. 4D). These results suggested that exosomal tRF-GluCTC promotes liver metastasis induced by PDAC derived exosomes.

The results of the collagen gel contraction assay and transwell migration assay showed that increased expression of tRF-GluCTC via exosome in p-HSCs and LX2 significantly enhanced cell contractility and migration ability (Fig. 4E, F). Under fluorescence microscopy, we found that the addition of PDAC-exo transfected with tRF-mimic resulted in increased cell volume and synapse formation in p-HSCs. Moreover, the fluorescence intensity of α-SMA and collagen I was also elevated (Fig. 4G). Furthermore, in both p-HSCs and LX2 cells, tRF-mimic upregulated both α-SMA and FAP, as well as the extracellular matrix protein. Conversely, the expression of these proteins was also downregulated in the tRF-inhibitor group (Fig. 4H).

### tRF-GluCTC promotes PDAC liver metastasis via WDR1

**Fig. 5:**
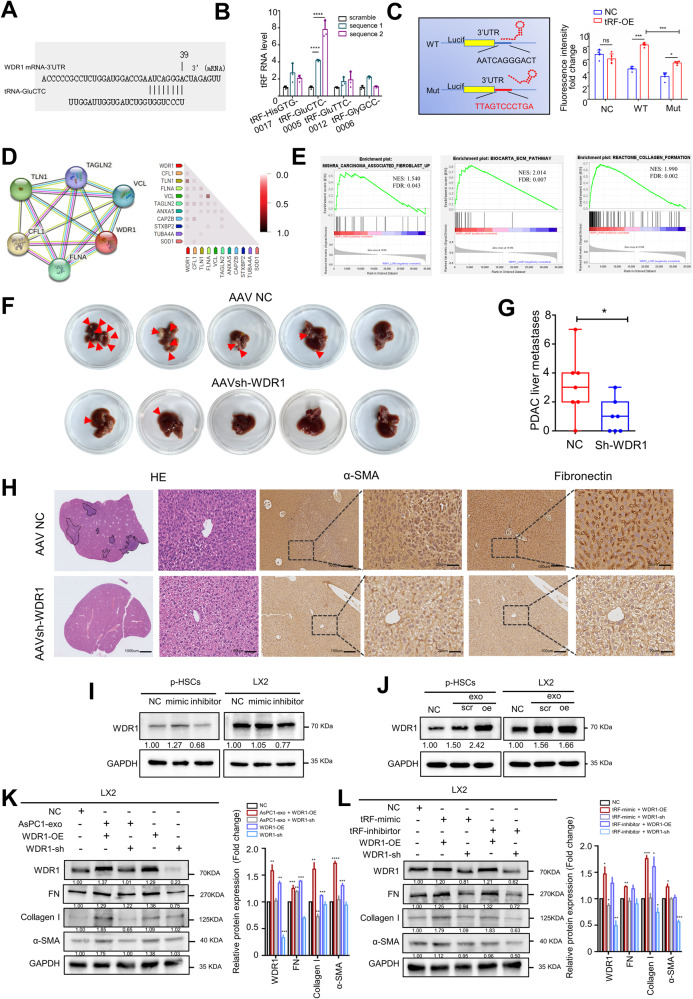
tRF-GluCTC promotes PDAC liver metastasis via WDR1. **A** The bioinformatics online (https://mirmap.ezlab.org) showed the binding targets of tRF-GluCTC in WDR1 mRNA. **B** RNA pull-down assay and RT-qPCR results showed that the only tRF interacted with WDR1 mRNA was tRF-GluCTC, while other candidate tRFs did not bind to it. **C** The schematic procedure of the luciferase reporter assay in 293 T cell. The results showed that a direct interaction might exist between tRF-GluCTC and WDR1 mRNA. **D** STRING (https://cn.string-db.org) was used to analyze the set of proteins associated with WDR1, and the correlation between WDR1 and cytoskeleton-associated proteins was significant. **E** GSEA for Cancer-associated fibroblast, ECM, and collagen formation. NES and false discovery rate (FDR) q value < 0.05. **F** Representative images of PDAC-liver metastasis foci. The red arrow shows the location of metastases from PDAC liver metastases between the AAV-NC group and the AAVsh-WDR1 group. **G** Statistics on the number of metastases from PDAC liver metastases. **H** Representative images of PDAC-liver metastasis tissue by IHC. Scale bar, 20 μm (left), 50 μm (right). **I** Western blot analysis of the expression of WDR1 in LX2 cells and p-HSCs after transfected the tRF-GluCTC mimic and inhibitor, **J** and after stimulated by the exosome transfected the tRF-GluCTC mimic or scramble. Scr: scramble. **K**, **L** The effect of WDR1 expression changes on PDAC-derived exosomes or the tRF-GluCTC in exosomes stimulated the LX2 activation. Each experiment was performed three times independently and the results are presented as mean ± SD. Student’s t-test was used to analyze the data; *p < 0.05, ***p < 0.001.

In order to investigate the mechanism by which exosomal tRF-GluCTC promotes liver metastasis in PDAC, we utilized bioinformatics analysis through the online tool mirmap to identify the binding targets of tRF-GluCTC. We found that the 3’ untranslated region (UTR) of WDR1 mRNA contains a sequence complementary to tRF-GluCTC, spanning eight bases (Fig. 5A).

To confirm the direct interaction between tRF-GluCTC and WDR1 mRNA, we applied an RNA pulldown assay with biotin-labeled WDR1 mRNA probe (Supplementary Fig. 5A). Interestingly, the results of qRT-PCR indicated a higher enrichment of tRF-GluCTC in the WDR1 mRNA probe group compared to the control probe group (Fig. 5B). The dual-luciferase reporter assay was also applied in 293 T cells. The full-length WDR1 mRNA-WT and a mutant version without tRF-GluCTC binding sites were subcloned into GP-miRGLO plasmids. The results indicated the luciferase activity of the WDR1 mRNA-WT group significantly increased in the tRF-GluCTC mimic group compared with the NC group. There was minimal difference between tRF-GluCTC mimics and the NC group in the luciferase activity of the WDR1 mRNA-Mut group (Fig. 5C). These results suggest a direct interaction between tRF-GluCTC and WDR1 mRNA in HSCs.

To further identify the potential pathways involved in HSC activation during WDR1-mediated facilitation of PDAC liver metastasis, we analyzed proteins associated with WDR1. The STRING analysis showed that the proteins associated with WDR1 are cytoskeleton-associated proteins (Fig. 5D). GSEA analysis was also performed in the PADC liver metastasis cohort from the Cancer Genome Atlas (TCGA) based on the transcriptional expression of WDR1. The results indicated the upregulation of ECM and collagen formation in the group with high WDR1 expression, which is known to be associated with PDAC liver metastasis (Fig. 5E).

In vivo, we employed an adeno-associated virus (AAV) to target interference with WDR1, and we observed a significant reduction in WDR1 expression in the liver of mice (Supplementary Fig. 4C, D). Subsequently, we injected 5*10^6^ Panc02 cells into the portal venous and observed the liver metastasis of PDAC after 6 weeks. The proportion of PDAC liver metastasis lesions was significantly smaller in the sh-WDR1 group compared to the NC group, and the size of the liver metastasis was also reduced (Fig. 5F, G). By immunohistochemical staining, we found that the fibrosis of the liver was obviously ameliorated in the sh-WDR1 group, accompanied by decreased expression of extracellular matrix proteins and HSC activation proteins (Fig. 5H).

To further confirm our finding, we transfected tRF-mimic and tRF-inhibitor in p-HSCs and LX2 cells, and found that tRF-GluCTC enhances the expression of WDR1, whereas the inhibitor of tRF-GluCTC represses WDR1 expression (Fig. 5I). Moreover, we added tRF-mimic-exo and tRF-scramble-exo in p-HSCs and LX2 cells. We observed an increase in WDR1 expression upon the addition of tRF-GluCTC (Fig. 5J). Subsequently, we added AsPC1-exo after regulating the expression of the WDR1 gene in LX2 cells. The results demonstrated that WDR1 could upregulate the expression of fibronectin and α-SMA in response to exosome stimulation, while the inhibition of WDR1 decreased the expression of these proteins (Fig. 5K & Supplementary Fig. 4E, F). Moreover, the ability of tRF-GluCTC to upregulate fibronectin and α-SMA was decreased after interfering with WDR1 expression. In contrast, overexpression of WDR1 rescued the inhibitory effect of tRF-inhibitor (Fig. 5L).

### WDR1 binds to YAP and promotes the Hippo signaling pathway in HSCs

**Fig. 6:**
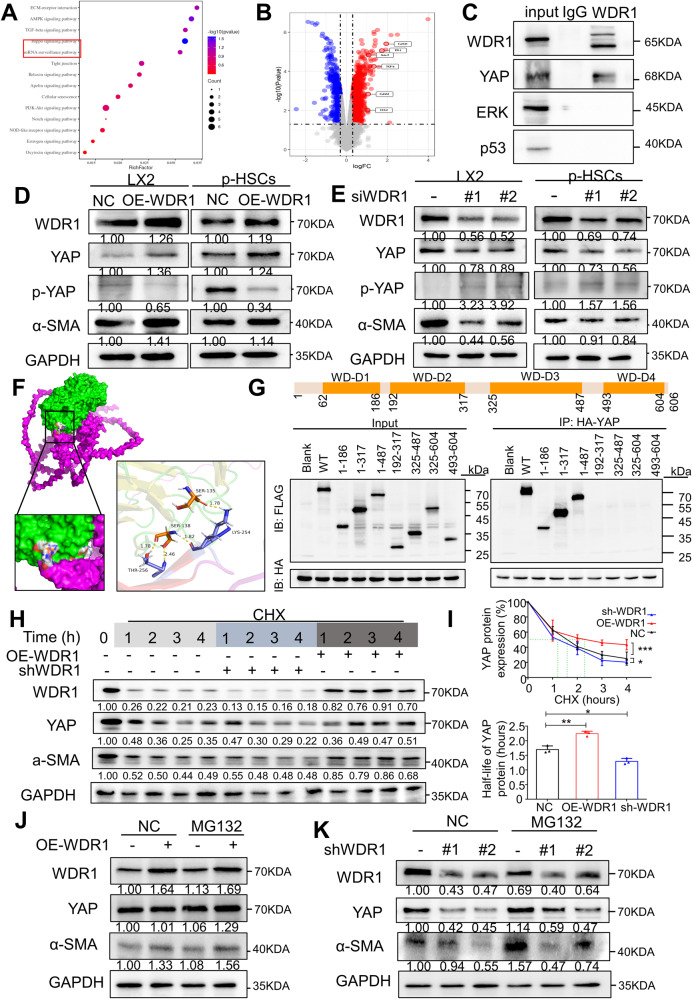
WDR1 binds to YAP and promotes the Hippo signaling pathway in HSCs. **A** The bubble plot represents pathway enrichment analysis performed on protein differentially upregulated in the interaction with WDR1 by Co-IP (log Fold-Change (FC) > 1). Reactome, Kyoto Encyclopedia of Genes and Genomes, and gene ontology pathways with adjusted *p* < 0.05 are displayed with the normalized enrichment score (NES) indicated on the x-axis. **B** Volcano plot of selected differentially regulated genes related to multiple ECM-related proteins in PDAC-exo treatment of HSCs vs untreated HSCs. The vertical dashed line indicates an adjusted *p* < 0.1, and the horizontal dashed lines indicate an absolute log2 FC < 1. **C** Reciprocal immunoprecipitation assays showed interaction of WDR1 with YAP. **D** Immunoblot analysis showed the expression of YAP and α-SMA after increased WDR1 and **E** interference WDR1 in LX2 cells and p-HSCs. **F** The 3D molecular docking results showed that the conformations of WDR1 and YAP protein targets contain good binding interactions. All the binding affinities lower than –5.0 kcal/ mol. **G** Truncation mapping of the WDR1-YAP binding domain. A schematic diagram shows the WDR1 protein domain structure. Immunoblot analysis shows FLAG-tagged full-length (WT) WDR1 and its truncated forms pulled down by HA-tagged YAP. **H** p-HSCs cells stably expressing WDR1 shRNA or WDR1 overexpression lentivirus were treated with or without cycloheximide (40 μg/mL), and harvested at the indicated times, protein levels of WDR1 and YAP were analyzed by immunoblotting. **I** Half-life statistics of YAP protein in p-HSCs under cycloheximide stimulation. **J**, **K** p-HSCs cells transfected with indicated siRNA or lentivirus were untreated or treated with MG132 (10 μmol/L) for 8 hours, followed by cell lysates being immunoblotted as indicated. Each experiment was performed three times independently and the results are presented as mean ± SD. Student’s *t*-test was used to analyze the data; *p < 0.05, **p < 0.01, ***p < 0.001.

It was reported that WDR1 encodes a protein containing 9 WD repeats, which are approximately 30- to 40-amino acid domains containing several conserved residues, mostly including a trp-asp at the C-terminal end. These WD domains are known to be involved in protein-protein interactions [41]. In the current study, aiming to clarify the potential regulatory mechanism of WDR promoting PDAC liver metastasis, the CoIP/MS technology was adopted to identify WDR1-interacting proteins in p-HSCs cells, and a total of 205 proteins were identified (Top 60 genes were shown in Supplementary Table S3). The overall profiles of the biological functions were analyzed for the candidate proteins via GO and pathway enrichment. The analysis revealed that WDR1 is involved in several cancer-associated pathways, including the AMPK signaling pathway, TGF-β signaling pathway, and Hippo signaling pathway (Fig. 6A). Meanwhile, the GEO RNA-seq database (GSE148139) showed increased expression of α-SMA and multiple ECM-related proteins after PDAC-exo treatment of HSCs (Fig. 6B).

Based on these findings, we next sought to validate the interaction of WDR1 and critical signaling pathway proteins using western blot assay repetitively. The western blot experiment showed that YAP was detected in the WDR1 pulled-down samples but not in the control group (Fig. 6C). This was further confirmed by interfering or overexpressing WDR1 in p-HSCs and LX2. The results revealed an increase in YAP expression and a decrease in YAP phosphorylation with WDR1 overexpression (Fig. 6D, E).

Next, we aimed to elucidate the crucial protein domains that are responsible for the interaction between WDR1 and YAP. Molecular docking was employed to investigate interactions between vital active compounds and major targets. The binding affinity lower than –5.0 kcal/ mol indicates that the conformations have good interactions. In this study, molecular docking results showed that the conformations of WDR1 and YAP protein targets contain good binding interactions, and the interactions were also reliable (Fig. 6F). Specifically, the residues SER135 and SER138 in the WDR1 protein were found to interact with the YAP protein. Based on their protein structures, truncated mutation plasmids of WDR1 [wild type (WT), WD-Domain1, WD-Domain2, WD-Domain3, and WD-Domain4] were constructed as indicated (Fig. 6G upper). CoIP experiments were conducted after transfecting these plasmids into 293 T cells. The result revealed that the deletion of the WD-Domain1 in WDR1 significantly disrupted the interaction (Fig. 6G lower). Additionally, the influence of the N-terminals of WDR1 in promoting the expression of a-SMA in p-HSCs cells was also investigated. To this end, exogenous N-terminals, M-terminals and C-tail of WDR1 were transfected into p-HSCs cells. The western blot assay showed that the expression of α-SMA was not increased in cells treated only with WDR1 M-terminals and C-tail, but it was enhanced significantly within the existing N-terminals and full-length WDR1 (Supplementary Fig. 4G).

To assess the role of YAP stability through WDR1 regulation, we manipulated the expression of WDR1 in p-HSCs cells and treated the cells with cycloheximide (CHX), a protein synthesis inhibitor. The half-life analyses revealed that YAP was more stable in WDR1-adequate cells (Fig. 6H, I). Since YAP was previously reported to be subjected to proteasomal degradation activated by upstream kinase, we sought to further confirm that WDR1 regulates the turnover of YAP. Upon treating WDR1-deficient cells with proteasome inhibitor MG132, we restored the protein levels of YAP which were previously decreased by WDR1 depletion. Also, the degradation process of YAP protein was significantly inhibited by adding MG132 in WDR1-adequate cells (Fig. 6J, K).

### tRF-GluCTC in PDAC-exo promotes MDSC infiltration in liver tissue

**Fig. 7:**
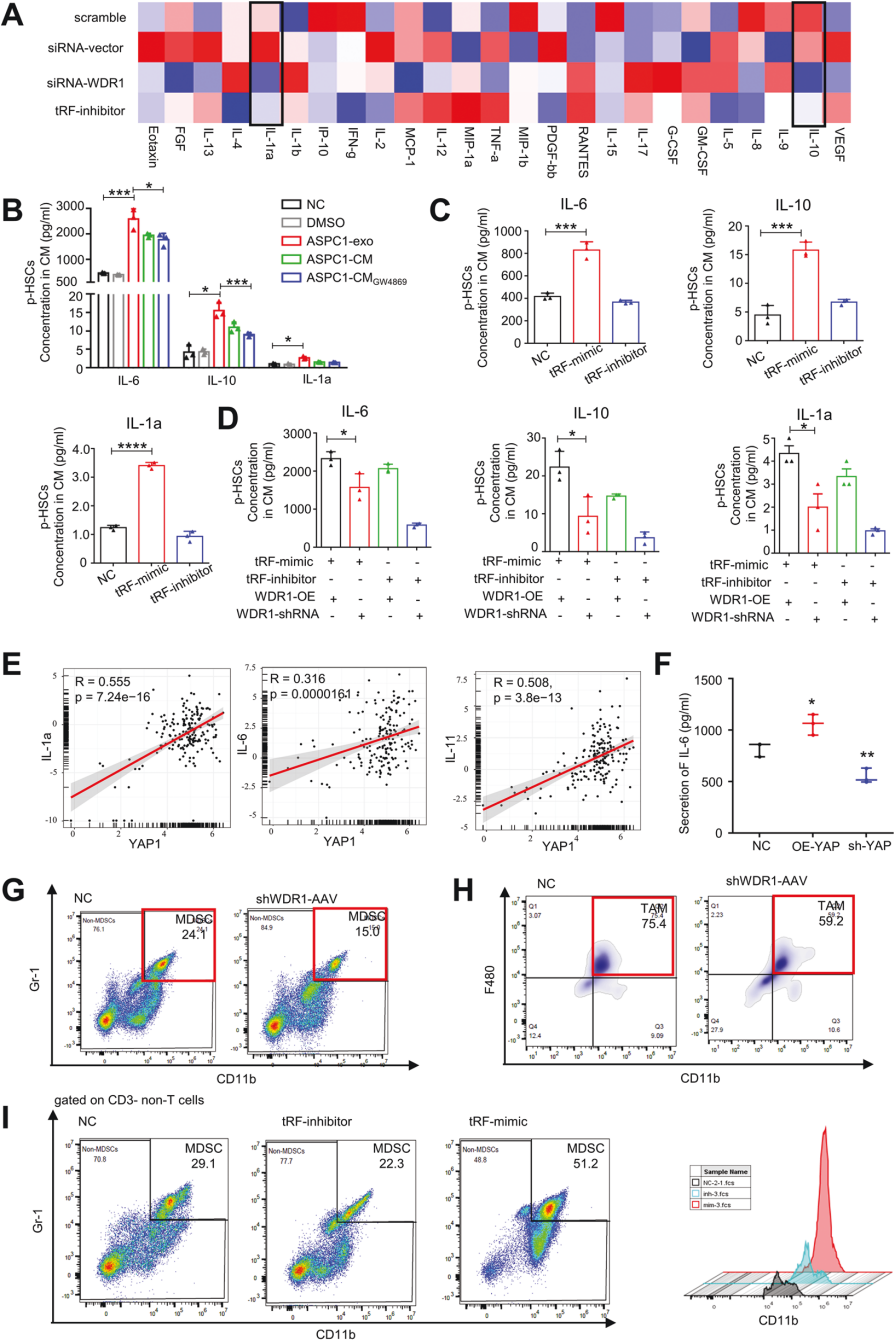
tRF-GluCTC in PDAC-exo promotes MDSC infiltration in liver tissue. **A** The heat map presentation showed the cytokines secretion changes by p-HSCs from WDR1 knockdown group and the tRF-GluCTC inhibitor-treated group. **B** Elisa assay results showed that IL-6, IL-10, and IL-1a secretion in p-HSCs CM was upregulated after ASPC1-exosome treatment. **C** Elisa assay results showed the expression of IL-6, IL-10, and IL-1a secretion in p-HSCs CM after tRF-GluCTC mimic or inhibitor transfection. **D** Elisa assay results showed the expression of IL-6, IL-10 and IL-1a secretion in p-HSCs CM after tRF-GluCTC and WDR1 regulated. **E** Pearson correlation analysis indicated that cytokines, such as IL-1a, IL-6 and IL-11 were positively correlated with YAP. **F** Elisa assay results showed that IL-6 secretion in p-HSCs CM was upregulated after YAP overexpression. **G** Analysis by flow cytometry showing changes in MDSC tumor-infiltrating immunosuppressive cells (CD11b+Gr-1+) and **H** the tumor-associated macrophage (CD11b + F4/80+) between NC group, and shWDR1-AAV group (*n* = 5 mice/arm). **I** Analysis by flow cytometry showing changes in MDSC between NC group, tRF-mimic or tRF-inhibitor transfected exosome treated group (*n* = 5 mice/arm).

The formation of a PMN is considered a crucial step in the establishment of a metastatic lesion. Previous studies have reported that exosomes derived from PDAC can promote the infiltration of MDSCs in the liver, leading to the formation of PMNs [42]. To explore the relationship between the tRF and the cytokines secreted by HSCs, we transfected WDR1 siRNA and tRF inhibitor into p-HSCs cells respectively, and analyzed the expression of various cytokines in the supernatants using protein microarrays. As compared to cells transfected with scramble RNA, p-HSCs which down-regulated WDR1 or the tRF-inhibitor decreased the release of IL-1a, and IL-10 (Fig. 7A). We also quantified the concentration of IL-6, IL-10, and IL-1a in the supernatant of p-HSCs after ASPC-1 derived exosome treatment using ELISA. The ASPC-1 exosome in p-HSCs significantly could increase the secretion of IL-6, IL-10, and IL-1a (Fig. 7B). To further investigate the stimulation of tRF-GluCTC in PMN immunosuppressive environment via cytokines, we quantified the concentration of IL-6, IL-10, and IL-1a in the supernatant of p-HSCs after regulation the expression of tRF-GluCTC. As expected, tRF-GluCTC can promote IL-6 and IL-1a release in p-HSCs (Fig. 7C). Furthermore, the expression of IL-6, IL-10, and IL-1a in the supernatant of p-HSCs is also up-regulated by the overexpression of WDR1 (Fig. 7D).

To explore the relationship between the transcriptional co-activator YAP and the cytokines secreted by HSCs, we analyzed the correlations among YAP and cytokines in TCGA data using the GEPIA website The results showed that YAP had positive correlations with IL-1a, IL-6, and IL-11 (Fig. 7E). We also quantified the concentration of IL-6 in the supernatant of p-HSCs after manipulating the expression of YAP using ELISA. Overexpression of YAP in p-HSCs significantly increased the secretion of IL-6 (Fig. 7F).

To investigate the role of WDR1 in promoting infiltration of MDSC within the liver, we harvested liver tissues from AAV-shWDR1 mice. Flow cytometry results revealed that interference with WDR1 expression significantly reduced MDSC infiltration in liver tissues (Fig. 7G), as well as decreased infiltration of macrophages (Fig. 7H). To determine whether CD11b^+^Gr1^+^ MDSCs infiltrate the liver in response to tRF stimulation in PDAC-exo, exosomes containing scramble RNA, tRF-mimic and tRF-inhibitor were injected into the peritoneal cavity of mice (Supplementary Fig. 5B). Flow cytometry analysis revealed that the tRF-GluCTC contained in PDAC-exo significantly increase the MDSC population in mouse liver tissue (Fig. 7I). Moreover, flow cytometry analysis revealed that tRF-GluCTC promoted the infiltration of macrophages in liver tissue (Supplementary Fig. 5C).

Overall, our results highlight the potential involvement of HSCs, specifically the upregulation of WDR1 and the secretion of cytokines like IL1, in the process of liver metastasis in pancreatic cancer. These findings contribute to a better understanding of the complex cellular interactions within the liver microenvironment during metastatic progression.

## Discussion

In this study, we investigated the role of tRF-GluCTC-0005 in the cargo of peripheral blood exosomes of PDAC patients that could assist in the diagnosis of PDAC liver metastasis. We further delved into the specific molecular mechanism of tRFs in promoting liver metastasis of PDAC. This study provided evidence that the tRFs positive exosomes secreted by PDAC cells play an important role in promoting liver metastasis of pancreatic cancer. The molecular mechanism study confirmed that tRF-GluCTC promotes the stability of WDR1 mRNA, preventing its degradation. The N-terminus of the WDR1 protein interacted with the YAP protein and further prevented YAP protein phosphorylation, thus promoting the secretion of cytokines cascades by HSCs resulting in the formation of hepatic PMNs.

With the development of sequencing technology, emerging evidence is revealing that the abnormal expression of tRFs is involved in the progression of human diseases, such as neurodegenerative diseases, inherited metabolic disorders, and cancers [43]. In our study, tRF-GluCTC-0005 caused overexpression of WDR1 protein by binding to the 3’ UTR of the mRNA of the WDR1 in HSCs, and inhibiting mRNA degradation. We identified the binding site of tRF-GluCTC-0005 and WDR1 mRNA using luciferase reporter assay in vitro, and used AAV to specifically interfere with the expression of WDR1 in the liver of mice in vivo. WDR1 is involved in the formation of liver fibrosis and provided favorable evidence for the view that tRF-GluCTC-0005 in PDAC derived exosomes promotes liver PMN formation through WDR1. Our study further expands our knowledge to delve into the roles of tRFs in cellular pathophysiological processes in both cancer tissue and other non-tumor tissues.

The detection of biomolecules in serum for clinical diagnosis and treatment is very important. In this study, we also found that the expression of tRF-GluCTC in the serum exosomes of patients with PDAC liver metastases was significantly higher than those without liver metastases. These results suggested that the expression of tRF-GluCTC in serum exosomes could be used as a predictor of pancreatic cancer liver metastasis. Additionally, it has been reported that there are abnormal alterations in the expression of tRFs in many kinds of tumor patients’ serum. Wu et al. identified that the profile of tRFs in the plasma of colorectal cancer (CRC) was significantly different from that of the healthy control group. This is particularly true for the expression of 5′-tRF and 5′-tRF-GlyGCC, whose levels are significantly increased in CRC plasma [43]. The clinicopathological data indicated that the levels of tRF-19-3L7L73JD showed diagnostic value in gastric cancer [44]. However, so far, there is no single stand-alone miRNA or tRF that has yet been identified as an ideal biomarker for the diagnosis of PDAC. Using a single exosome-derived biomolecule as an indicator to diagnose disease is still a challenge.

An increased degree of liver fibrosis can significantly contribute to the proportion of liver metastases from PDAC [45]. A study published by Zhang et al. showed that autophagy dysfunction and HSCs activating could promote hepatic fibrosis. Targeting the activation of HSCs is a promising strategy for treating fibroproliferative liver diseases [46]. The HSCs could be activated by various stimulations and secrete large amounts of extracellular matrix proteins, which promote CTCs colonization, thus facilitating the process of liver metastasis from PDAC [47]. In this study, we found that HSCs were significantly activated after the uptake of exosomes in vitro. Under fluorescence microscopy, we could observe a remarkable increase in the cell volume and synapses. The expression of HSCs activation protein (α-SMA) and cellular matrix protein (Fibronectin) in the cells was also increased. Furthermore, in vivo, we examined the extent of liver fibrosis in mice after tail vein injection of exosomes. By Masson staining, we found that the exosomes secreted by AsPC-1 cell line could promote liver fibrosis. We regulated the expression of tRF-GluCTC in exosomes, and the degree of liver fibrosis increased.

The infiltration of MDSCs promoting PMN formation during liver metastasis of PDAC is also an important influencing factor [48]. We found that MDSCs infiltration significantly promoted the hepatic metastatic process of PDAC by flow cytometric analysis of various types of immune cells. Analysis of single-cell sequencing data also showed that the proportion of MDSC was higher in the livers of mice that developed PDAC liver metastasis, and the infiltration of macrophages was also significantly enhanced.

As a protein that assists cofilin-mediated actin filament disassembly, WDR1 is overexpressed in several kinds of cancer [23, 49, 50]. We found that tRF-GluCTC could interact with the mRNA of WDR1 through bioinformatics technology, thus regulating the translation process of WDR1. Under the regulation of AAV interference of WDR1, a significant reduction in metastatic foci in the liver and a corresponding reduction in liver fibrosis in a mouse PDAC liver metastasis model has been detected. These results suggest that WDR1 can promote the activation of HSCs and facilitate the process of liver metastasis of pancreatic cancer. In addition, we deeply investigated the interaction of structural domains between WDR1 and YAP in HSCs. We separately involved a variety of WDR1 proteins with different truncations and detected their interaction with YAP using COIP. The results showed that only the N-terminal of WDR1 could bind to YAP, while the other truncated proteins did not bind to each other. 3D molecular docking prediction results also confirmed this idea. These experiments have revealed the precise interaction sites between WDR1 and YAP proteins for the first time, elucidating the interaction relationship between the two important intercellular key signaling molecules.

Despite the advances provided by this study, several limitations should be acknowledged. Firstly, while tRF-GluCTC in serum exosomes showed diagnostic potential in the PDAC liver metastasis cohorts, relying solely on a single RNA expression as an indicator carries risks. Combining tRF-GluCTC with other known biomacromolecules would enhance the diagnostic value. Secondly, although the study examined the effects of tRF-GluCTC on IL-6 expression, the roles of other cancer-related cytokines remain unknown. Exploring the involvement of additional cytokines produced by HSCs in PDAC liver PMNs would be valuable. Thirdly, future research should focus on exploring the crosstalk between tumor cells and pre-metastatic niche microenvironment cells of distant metastases using organoids to simulate the internal environment.

In conclusion, this study provides insights into the clinical diagnosis and molecular mechanism of tRFs in promoting liver metastasis of PDAC. Our findings suggest that tRF-GluCTC in serum exosomes could serve as a predictor for PDAC liver metastasis. This study also highlights the role of tRF-GluCTC in regulating WDR1 and its implications in HSC activation and liver fibrosis, and the molecular interactions in HSCs are expected to provide clues for subsequent target therapy.

### Supplementary information

supplementary figure 1

supplementary figure 2

supplementary figure 3

supplementary figure 4

supplementary figure 5

supplementary legends

Supplementary Table

Original Data File

aj-checklist

## Data Availability

All data that support the findings in this study are available from the corresponding author upon reasonable request.
