# Supplementary figures and images for: Exosome-derived tRNA fragments tRF-GluCTC-0005 promotes pancreatic cancer liver metastasis by activating hepatic stellate cells
Source: Cell Death Dis. 2024 Jan 30;15(1):102. doi: 10.1038/s41419-024-06482-3 (PMC10827722; doi:10.1038/s41419-024-06482-3)

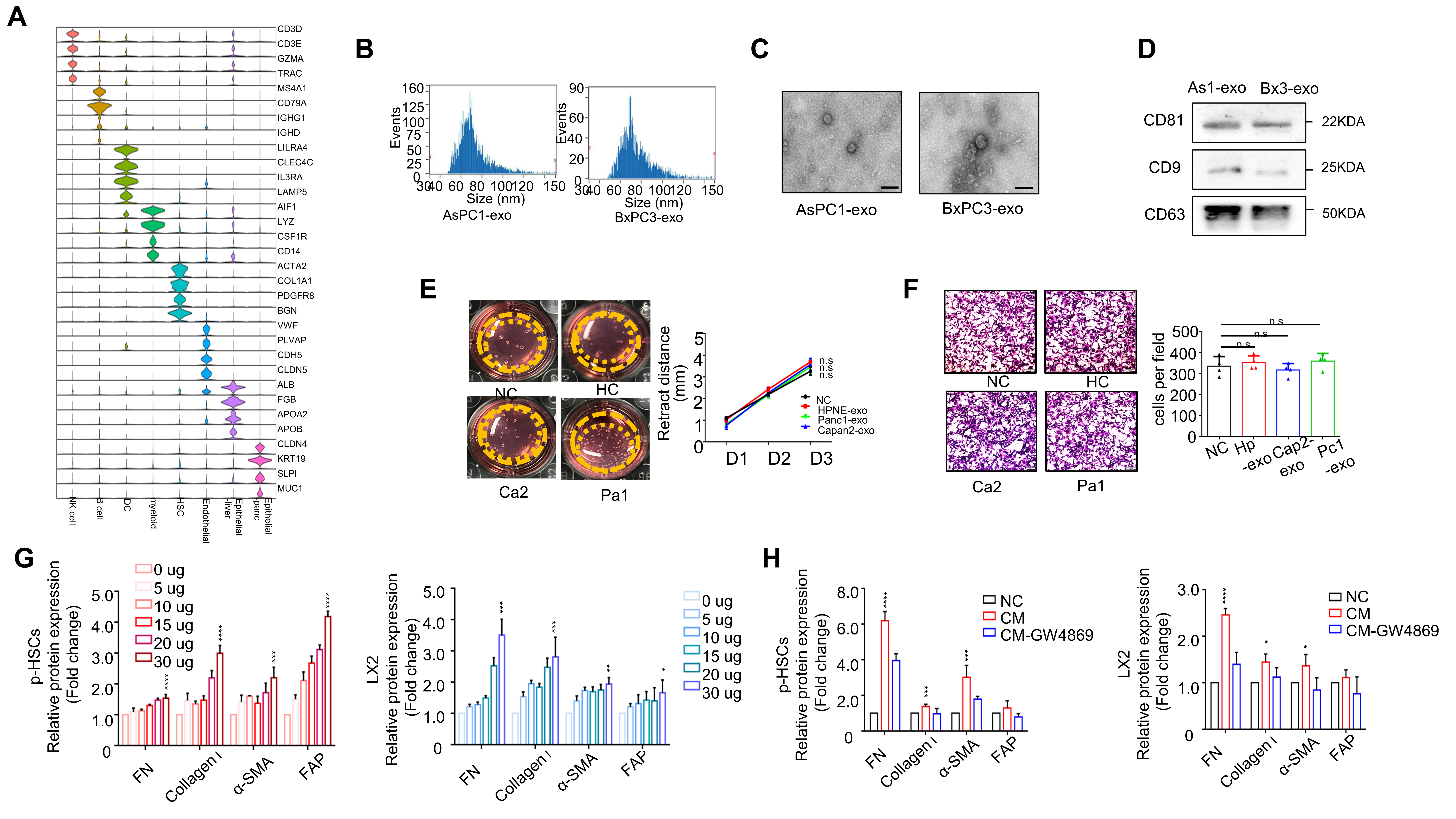

Supplement: Supplementary file 1 — supplementary figure 1 [file 41419_2024_6482_MOESM1_ESM.tif]

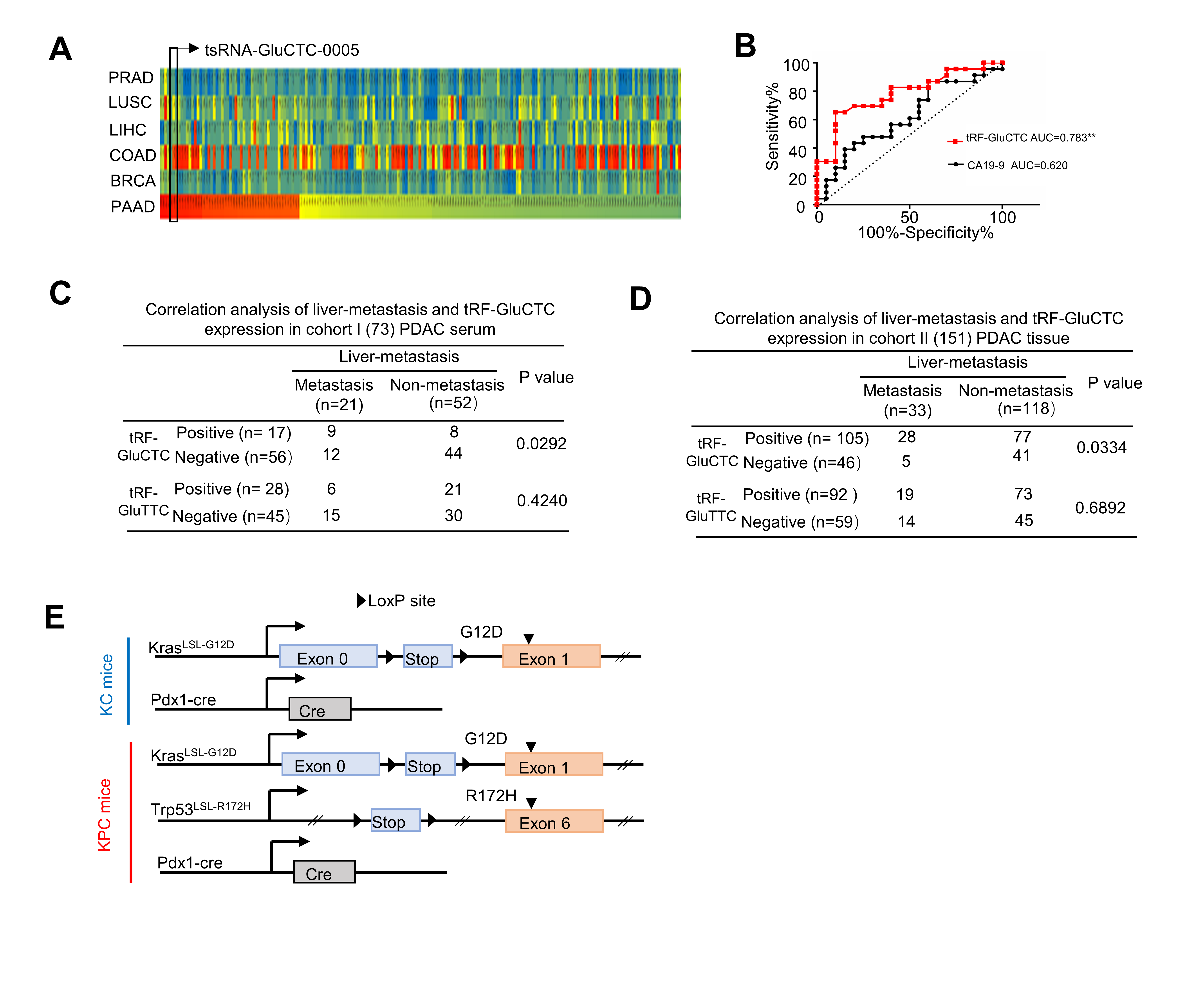

Supplement: Supplementary file 2 — supplementary figure 2 [file 41419_2024_6482_MOESM2_ESM.tif]

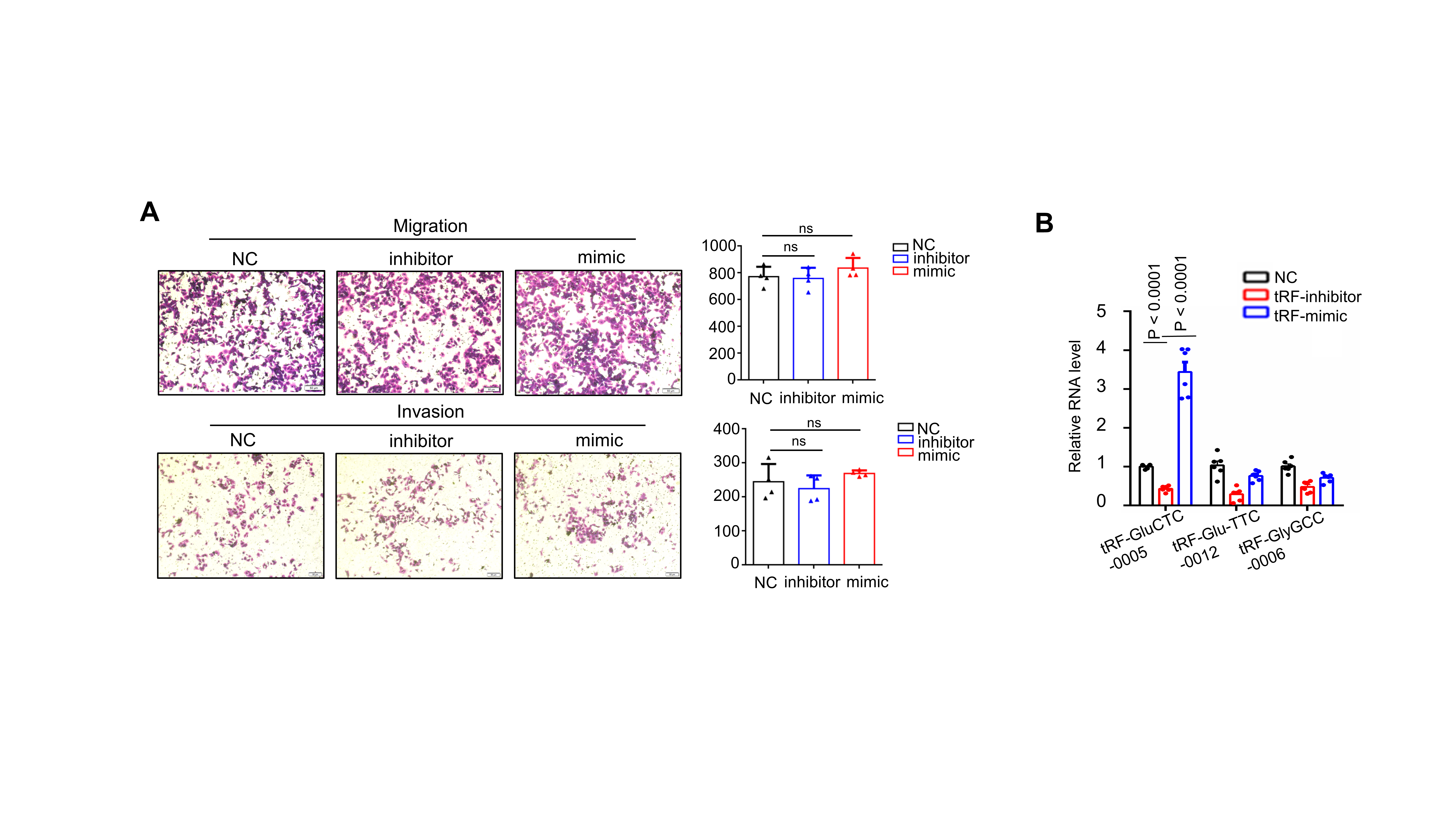

Supplement: Supplementary file 3 — supplementary figure 3 [file 41419_2024_6482_MOESM3_ESM.tif]

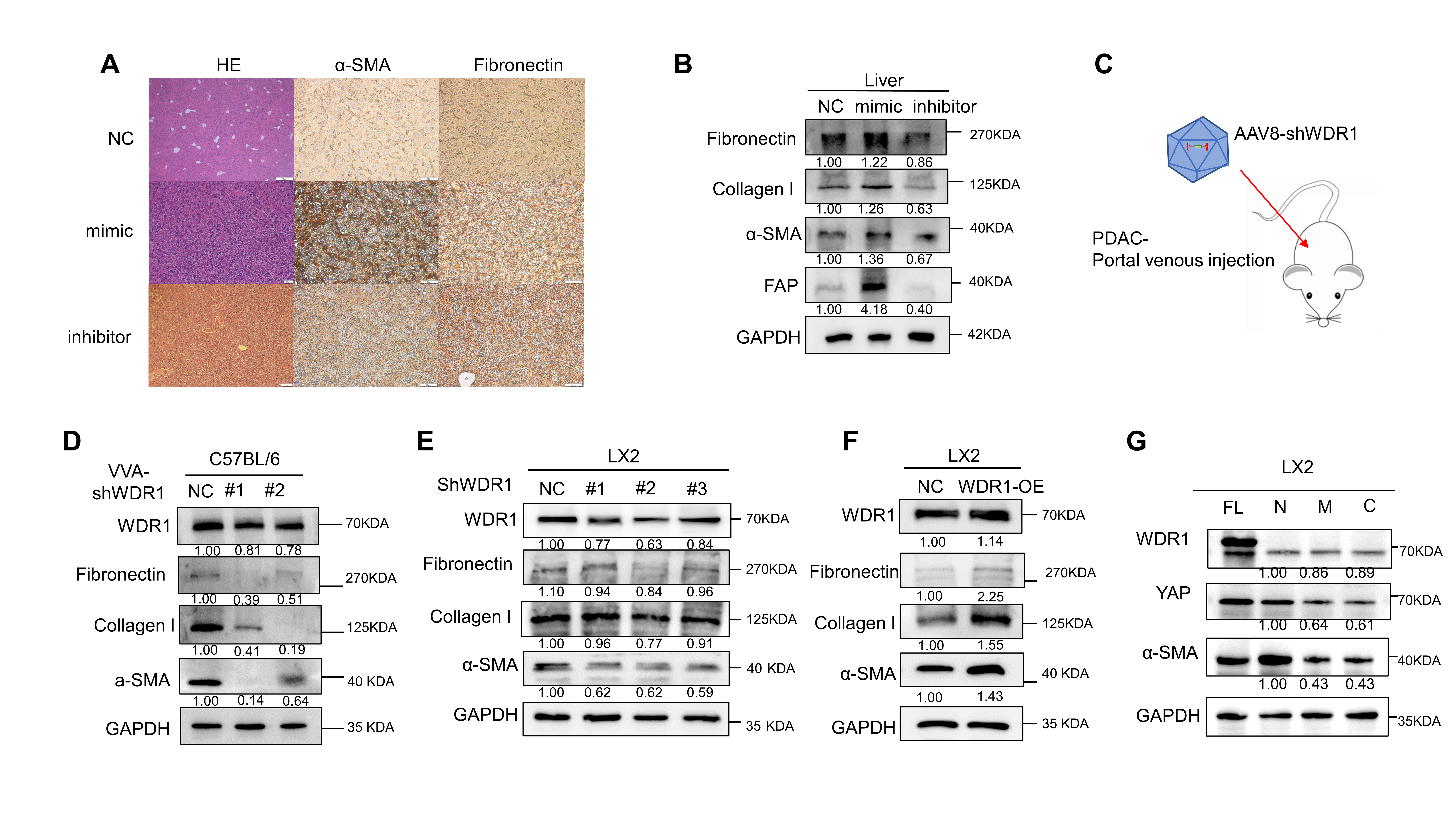

Supplement: Supplementary file 4 — supplementary figure 4 [file 41419_2024_6482_MOESM4_ESM.tif]

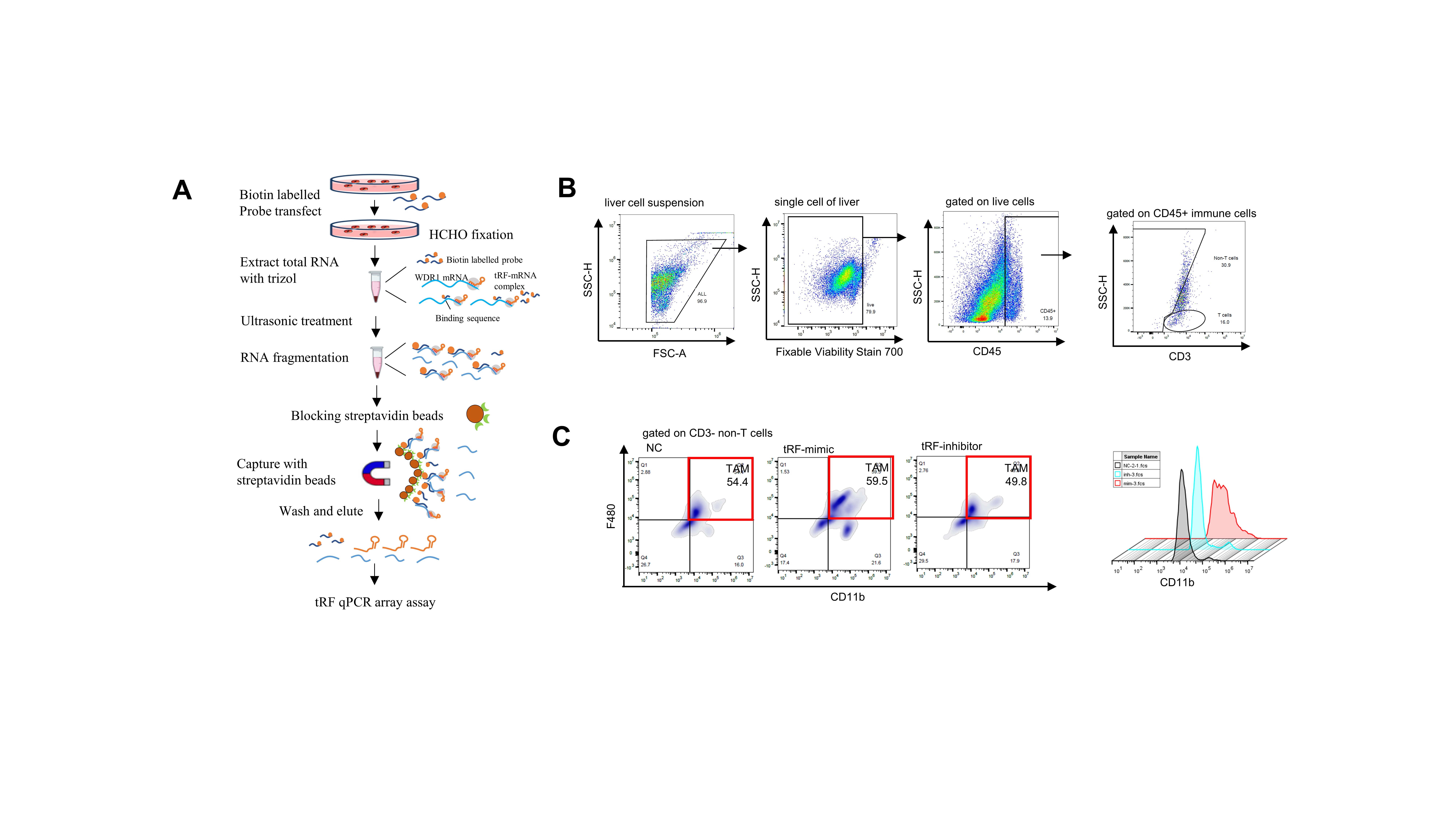

Supplement: Supplementary file 5 — supplementary figure 5 [file 41419_2024_6482_MOESM5_ESM.tif]

Fig2

H

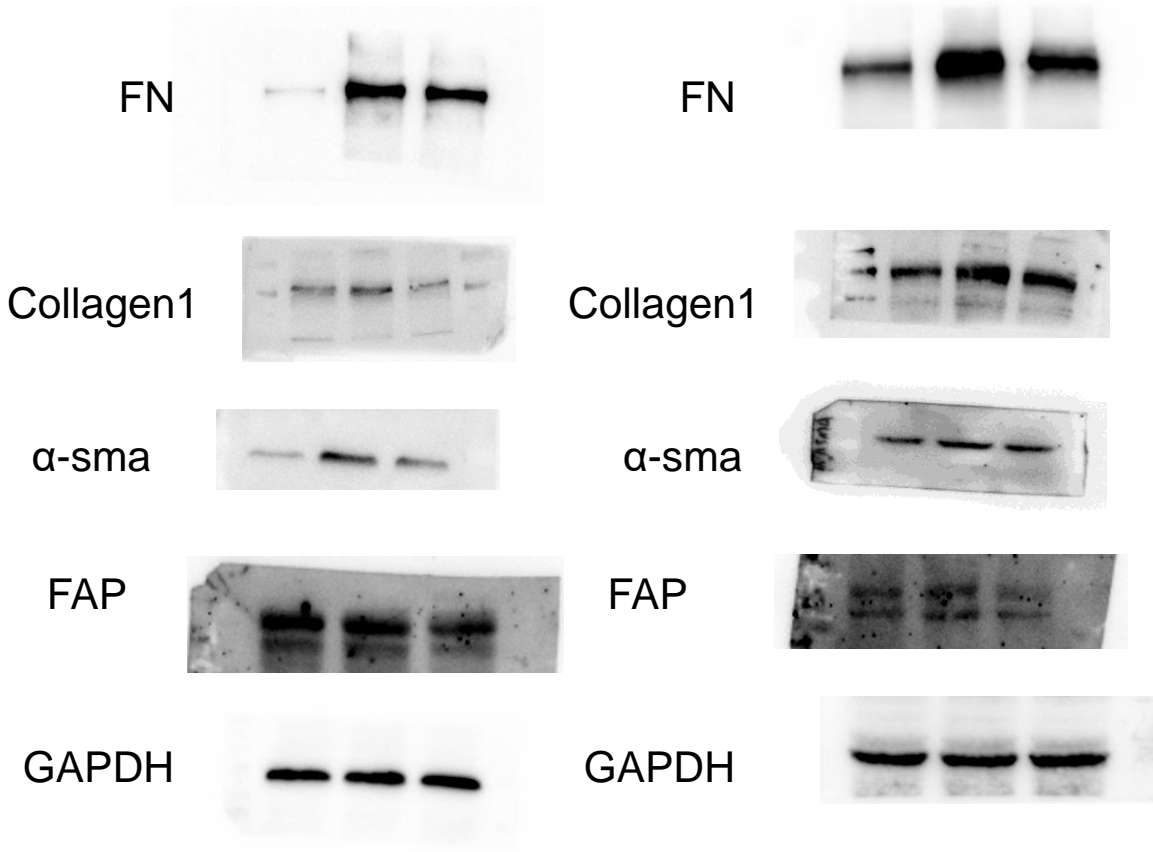

I

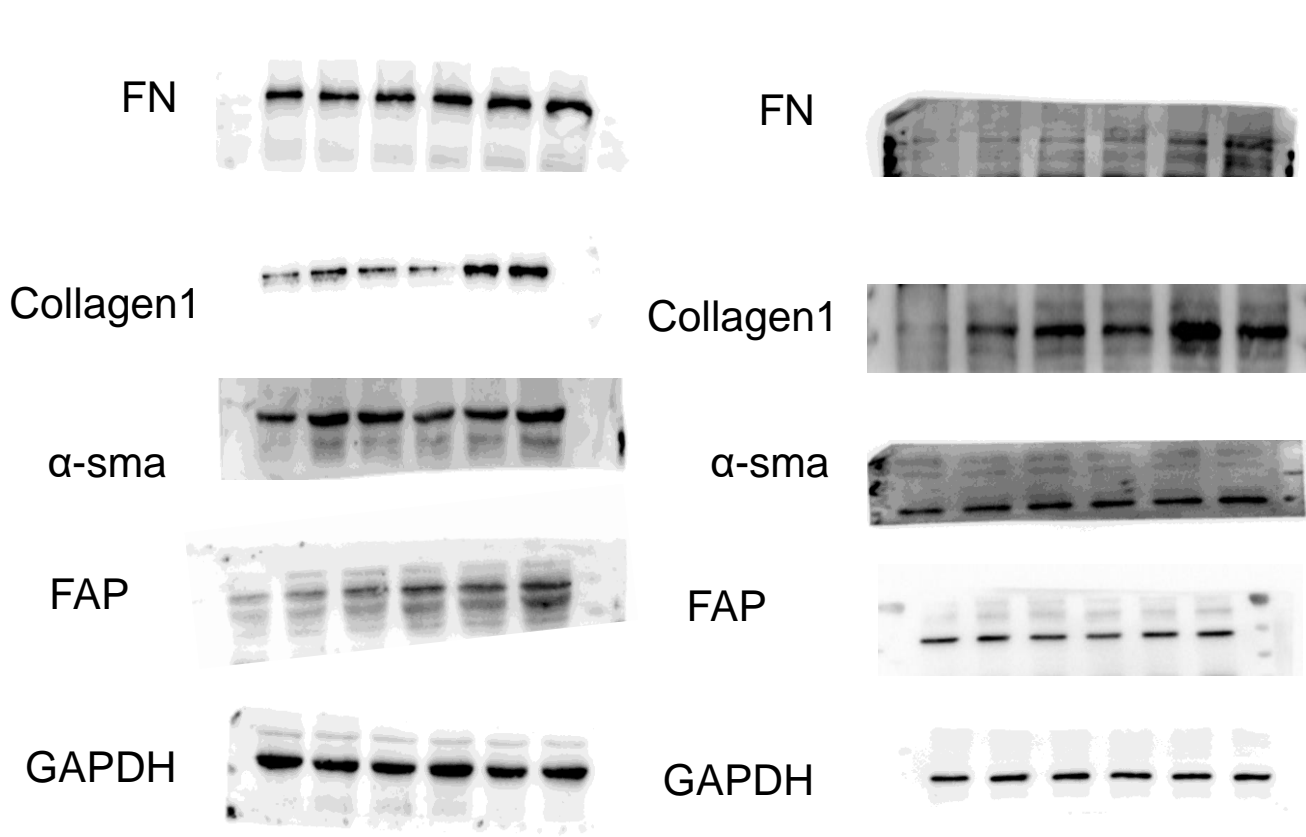

Fig4

H

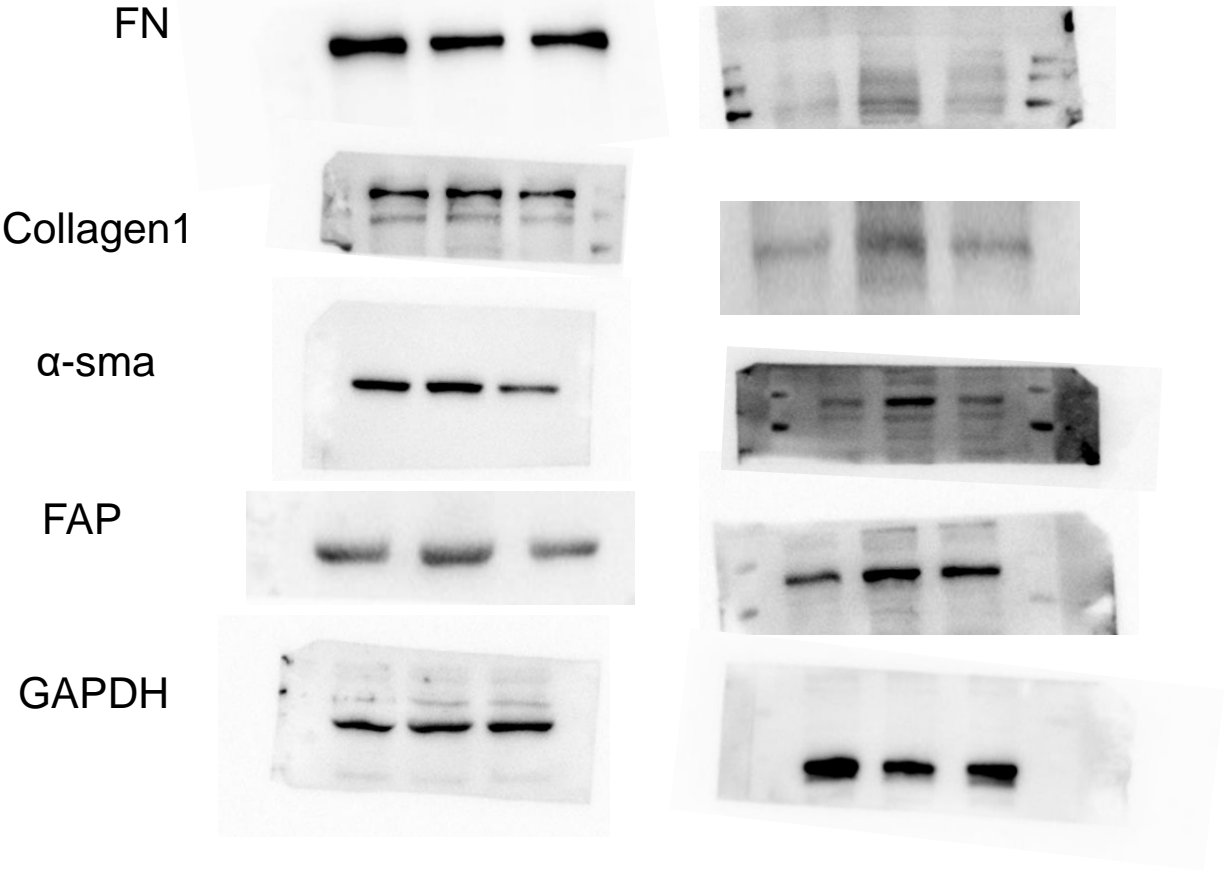

Fig5

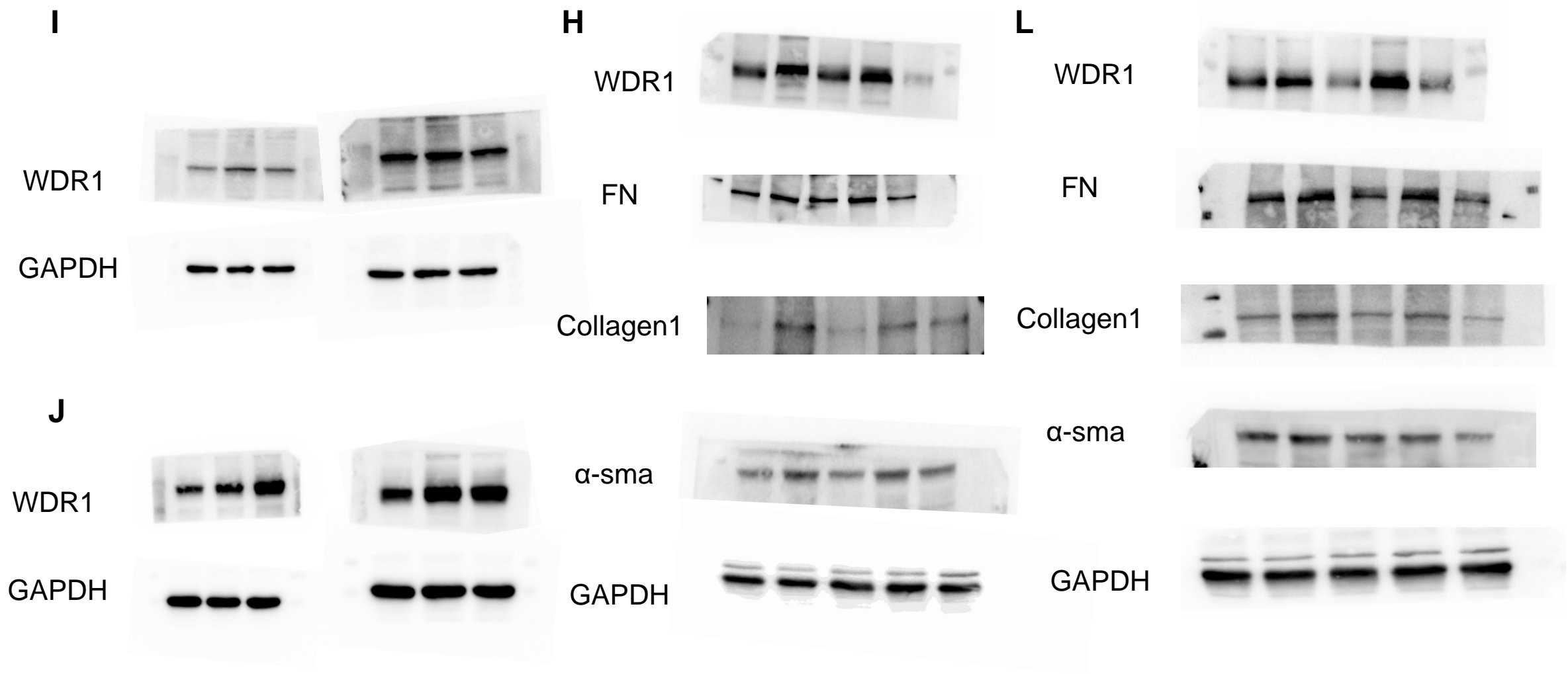

Fig6

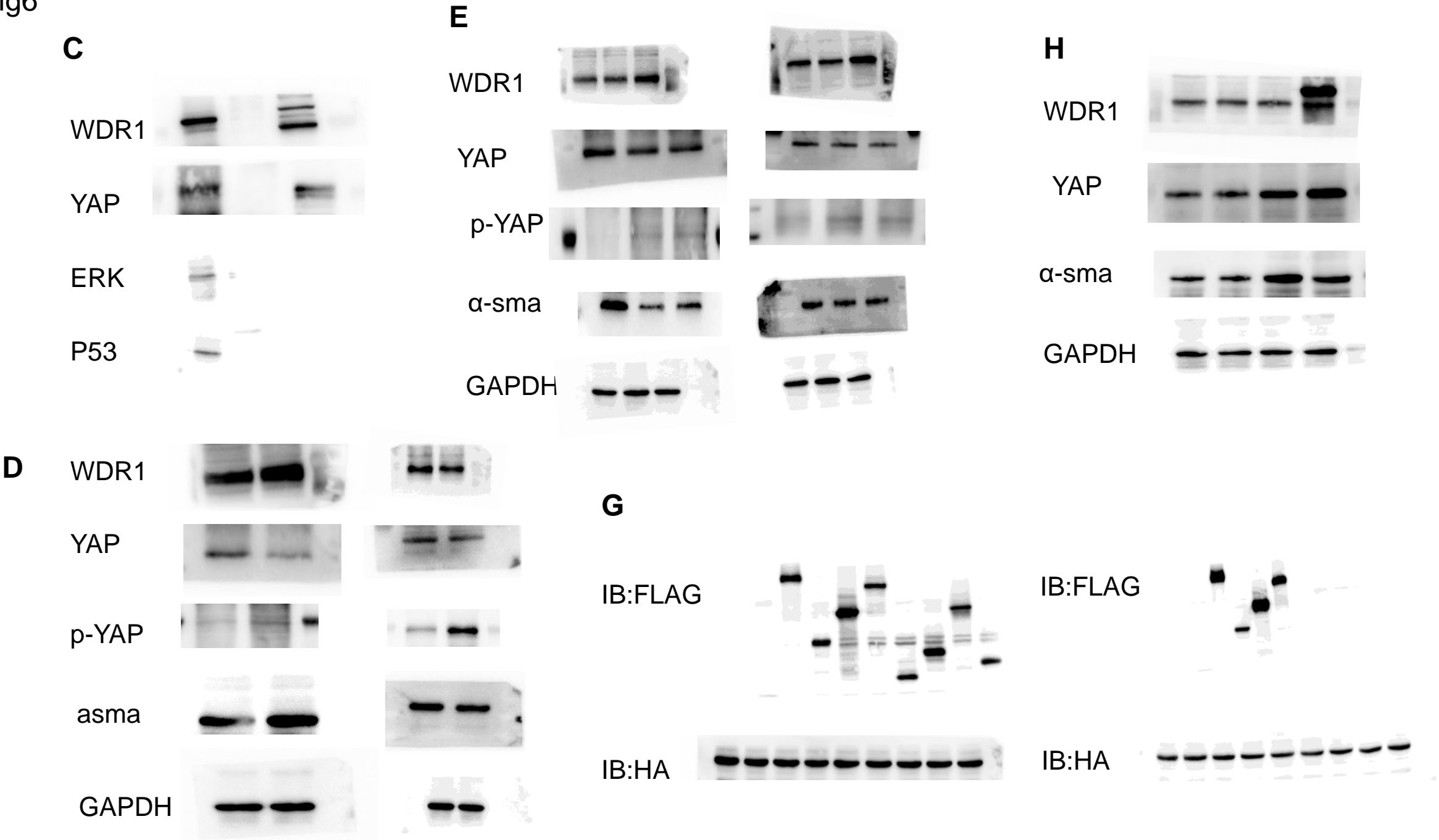

Fig6

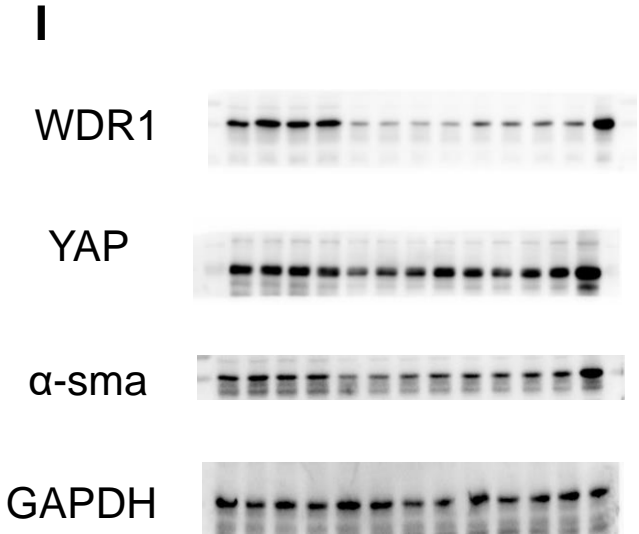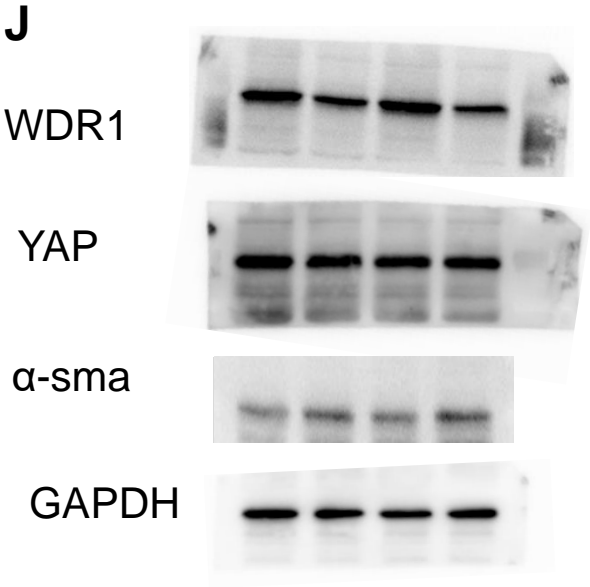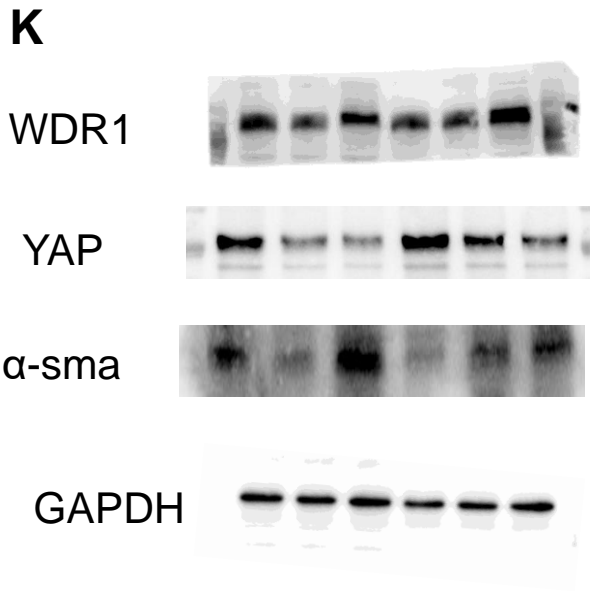

**C**

CD81

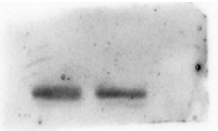

CD9

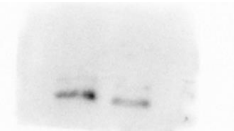

CD63

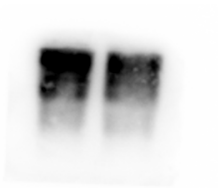

**D**

FN

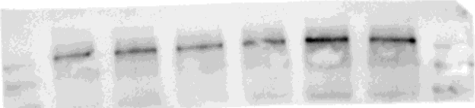

Collagen1

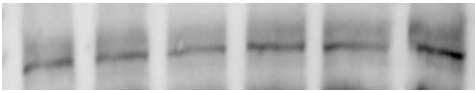

$\alpha$ -sma

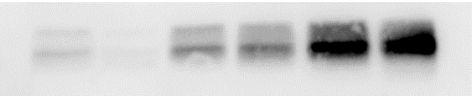

FAP

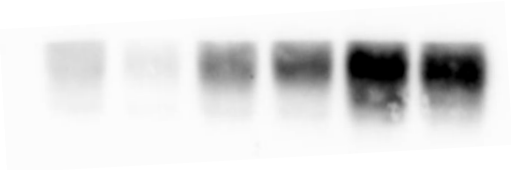

GAPDH

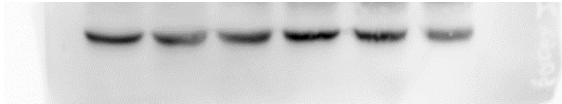

**B**

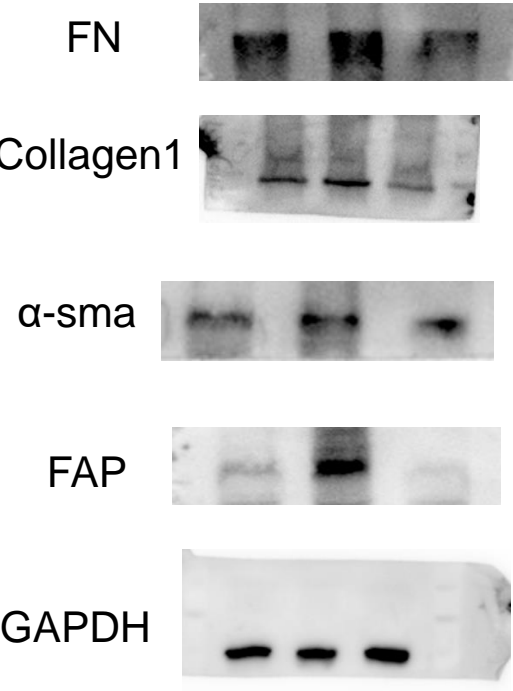

**C**

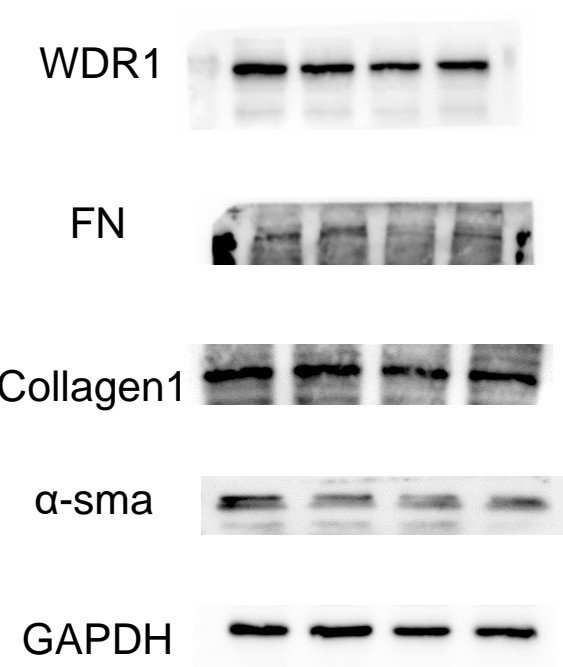

**D**

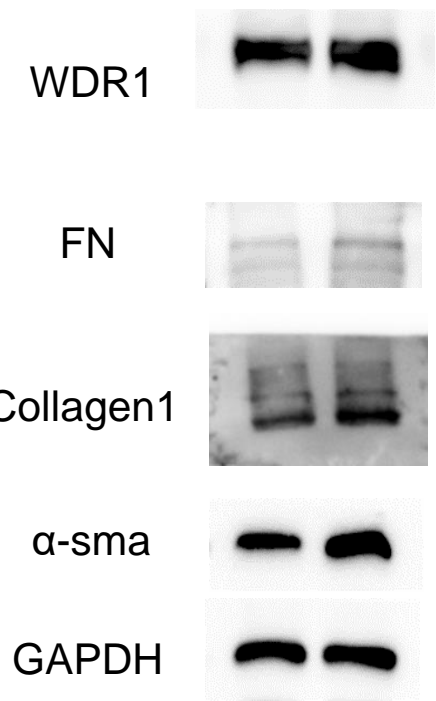

**F**

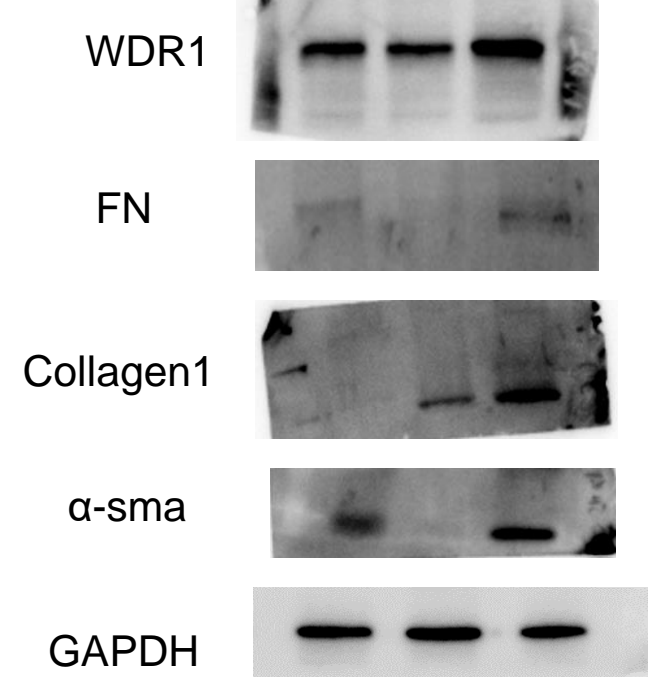

Supplement: Supplementary file 8 — Original Data File [file 41419_2024_6482_MOESM8_ESM.pdf]
