# Supplementary material for: Exosome-derived tRNA fragments tRF-GluCTC-0005 promotes pancreatic cancer liver metastasis by activating hepatic stellate cells
Source: Cell Death Dis. 2024 Jan 30;15(1):102. doi: 10.1038/s41419-024-06482-3 (PMC10827722; doi:10.1038/s41419-024-06482-3)
Supplement: Supplementary file 6 — supplementary legends [file 41419_2024_6482_MOESM6_ESM.docx]

**Sup Figure 1:** (A) violin plot depicts the expression level of selected genes expressed in each single cell. (B) Exosomes from AsPC1 and BxPC3 cell culture supernatant were analyzed by electron microscopy and (C) LM10 nanoparticle characterization system. Scale bar, 200 nm. (D) Immunoblot analysis showed the expression of exosomal characteristic proteins, CD9, CD81, and CD63. (E) Collagen gel containing p-HSCs was treated with exosomes from different cell lines of PDCA 10ug per well or without treatment (NC). Representative collagen gel images from 3 independent experiments are shown. Statistical significance relative to the NC group. (F) Transwell migration assays showed the migration abilities in p-HSCs stimulated by exosomes from different cell lines of PDCA. Scale bar, 100 μm. (G) Statistical table of differences in expression of related proteins under different exosome concentration treatments by western blot assay. (H) Statistical table of differences in expression of related proteins under different treatments by western blot assay.

**Sup Figure 2:** (A) The expression of tRFs in different tumor tissues in tdRFun (https://rna.sysu.edu.cn/tsRFun/index.php). PRAD: Prostate adenocarcinoma, LUSC: Lung squamous cell carcinoma, LIHC: Liver hepatocellular carcinoma, COAD: Colon adenocarcinoma, BRCA: Breast invasive carcinoma, PAAD: Pancreatic adenocarcinoma. (B) ROC analysis of the individual exosomal tRF-GluCTC and the CA19-9 for PDAC patients and non-cancerous control. (C&D) Correlation between the PDAC liver metastasis and tRF-GluCTC or tRF-GluTTC expression in peripheral blood serum exosomes was shown in two cohorts. (E) Schematic diagram of the conditional expression strategy of Kras-G12D and Trp53-R17H mutant gene in KPC mice.

**Sup Figure 3:** (A) Transwell migration assays showed the migration abilities in AsPC1 transfected with mimics and inhibitors of tRF-GluCTC. Scale bar, 100 μm. (B) RT-qPCR results of the tRF-GluCTC in exosomes from AsPC1 transfected with mimic and inhibitor. The expression of tRF-GluCTC was altered, while the expression of other tRFs remained unaffected.

**Sup Figure 4:** (A) Representative images of tRF-mimic and tRF-inhibitor exosome stimulated mice liver tissue by IHC. Scale bar, 20 μm (HE), 50 μm (IHC). (B) Immunoblot analysis showed the expression of α-SMA and FAP was higher in the tRF-mimic group, and similarly the expression of extracellular matrix proteins. (C) Schematic diagram of AAV mediated sh-WDR1 expression in mice liver. (D) Immunoblot analysis showed a significant reduction in WDR1 expression in the liver of mice disturbed with adeno-associated virus (AAV) target with WDR1. (E & F) Immunoblot analysis showed that WDR1 could upregulate the expression of fibronectin and α-SMA in response to exosome stimulation, while the inhibition of WDR1 decreased the expression of these proteins. (G) Immunoblot analysis showed the expression of YAP and α-SMA after regulating the WDR1 protein domain structure in LX2 cells.

**Sup Figure 5:** (A) The schematic procedure of probing the binding tRFs of WDR1 mRNA in 293T cells by RNA pull-down assay. (B) MDSC cells isolated by flow cytometry of C57BL/6 mice. Fixable viability stain 700 and the pan leukocyte marker CD45 were used to eliminate potential contamination by cell debris or non-hematopoietic progenitor cells, respectively. (C) Analysis by flow cytometry showing changes in the tumor-associated macrophage (CD11b+F4/80+) between NC group, tRF-mimic or tRF-inhibitor transfected exosome treated group (n = 5 mice/arm).
