# Supplementary material for: Exosome-derived tRNA fragments tRF-GluCTC-0005 promotes pancreatic cancer liver metastasis by activating hepatic stellate cells
Source: Cell Death Dis. 2024 Jan 30;15(1):102. doi: 10.1038/s41419-024-06482-3 (PMC10827722; doi:10.1038/s41419-024-06482-3)
Supplement: Supplementary file 7 — Supplementary Table [file 41419_2024_6482_MOESM7_ESM.docx]

**Table S1.**

Sequences of siRNAs or mimics used in this study.

siRNAs Sequence (5’ → 3’)

| siControl | UUCUCCGAACGUGUCACGUTT |
| --- | --- |
| siWDR1#1 | GTCAAGGAGTGGACAATCA |
| siWDR1#2 | GCGGCAAGTCCTACATTTA |
| siWDR1#3 | GTCACAGTAAATCGATCCA |
| siYAP#1 | GACCAATAGCTCAGATCCTTT |
| siYAP#2 | GCCACCAAGCTAGATAAAGAA |
| siYAP#3 | CAGGTGATACTATCAACCAAA |
| scramble | TTCTCCGAACGTGTCACGT |

Mimics and Inhibitor Sequence (5’→ 3’)

| *tRF-GluCTC-0005* Control | UUGUACUACACAAAAGUACUG |
| --- | --- |
| *tRF-GluCT-0005* mimics | UCCCUGGUGGUCUAGUGG |
| *tRF-GluCT-0005* inhibitor | CCACUAGACCACCAGGGA |

Primers, probes and adaptor used in this study

FL-PCR Sequence and Primers for alternative splicing Sequence (5’ → 3’)

| WDR1-F primer | cgggatccgccaccatgccgtacgagatcaagaaggtgttcgcca |
| --- | --- |
| WDR1-R primer | ccctcgaggtaggtgattgtccactccttgacagaggca |
| YAP-F primer | cgggatccgccaccatggatcccgggcagcag |
| YAP-R primer | ccctcgagctgagctgtgggtgtagctg |
| WDR1-M1-F primer | cgacgcgtgccaccgaccacagccgctttgtcaactgtg |
| WDR1-M1-R primer | ccctcgagcttgatgacgtgcaggggctt |
| WDR1-M2-F primer | cgacgcgtgccaccggtcacagtaaatcgatccagtgtctgac |
| WDR1-M2-R primer | ttgcggccgccctctaggagcttgccctcatccttc |
| WDR1-C1-F primer | cgggatccgccaccatgggtcacagtaaatcgatccag |
| WDR1-C1-R primer | ccctcgaggtaggtgattgtccactccttgacagaggca |
| WDR1-C2-F primer | cgggatccgccaccatggccaagggccccgtgaccgacgtggcctact |
| WDR1-C2-R primer | ccctcgaggtaggtgattgtccactccttgacagaggca |
| WDR1-N1-F primer | cgggatccgccaccatgccgtacgagatcaagaaggtgttcgcca |
| WDR1-N1-R primer | ttgcggccgccgccaattgtgaacttgaacttgaatgggggtccct |
| WDR1-N2-F primer | cgggatccgccaccatgccgtacgagatcaagaaggtgttcgcca |
| WDR1-N2-R primer | ccctcgagcttgatgacgtgcaggggctt |
| WDR1-N3-F primer | cgggatccgccaccatgccgtacgagatcaagaaggtgttcgcca |
| WDR1-N3-R primer | ccctcgagctctaggagcttgccctcatcct |

RNA pull down probes (3’-biotin) 0Sequence (5’ → 3’)

| antisense ChIRP NC_1 | TCTGTCAAGGAGTGGACAAT- /3bio/ |
| --- | --- |
| WDR1 probe#1 | ATTGTCCACTCCTTGACAGA- /3bio/ |
| WDR1 probe#2 | GGATCTTGACTCTGGTTTCC- /3bio/ |

Reverse transcription primers for tRFs Sequence (5’ → 3’)

| tRF-HisGTG- primer | GTCGATCGACTGCAGGGTCCGAGGTATTCGCAGTCGATACGACCTAACC |
| --- | --- |
| tRF-GlyGCC-primer | GTCGTATCGACTGCAGGGTCCGAGGTATTCGCAGTCGATACGACTTCTAC |
| tRF-GluTTC- primer | GTCGTATCGACTGCAGGGTCCGAGGTATTCGCAGTCGATACGACGCCGAA |
| tRF-GluCTC- primer | GTCGTATCGACTGCAGGGTCCGAGGTATTCGCAGTCGATACGACTAGTGG |

qRT-PCR primers for tRFsSequence (5’ → 3’)

| Common F primer | ACTGCAGGGTCCGAGGTATT |
| --- | --- |
| tRF-HisGTG-R primer | GGCGCCGTGATCGTATAGT |
| tRF-GlyGCC-R primer | CGCATGGGTGGTTCAGTG |
| tRF-GluTTC-R primer | CGGCTGGTCTAGTGGCTAGGA |
| tRF-GluCTC-R primer | ACTAGACCACCAGGGATT |

Gene Symbol Sequence (5’ → 3’)

| gene | Forward | Reverse |
| --- | --- | --- |
| WDR1 | GGGATACCACGCAGAAGGAG | GCTTGATGTCCACGCTGTT |
| MMP9 | AGACCTGGGCAGATTCCAAAC | CGGCAAGTCTTCCGAGTAGT |
| IL-10 | GACTTTAAGGGTTACCTGGGTTG | TCACATGCGCCTTGATGTCTG |
| MMP2 | TACAGGATCATTGGCTACACACC | GGTCACATCGCTCCAGACT |
| a-SMA | CTATGAGGGCTATGCCTTGCC | GCTCAGCAGTAGTAACGAAGGA |
| Fibronectin | CGGTGGCTGTCAGTCAAAG | AAACCTCGGCTTCCTCCATAA |
| COL1A1 | GTTGCTGCTTGCAGTAACCTT | AGGGCCAAGTCCAACTCCTT |

**Table S2.**

Antibodies used in this study

| Protein name | Host species | Product number | dilutions |
| --- | --- | --- | --- |
| WB antibody | | | |
| a-SMA | M | BOSTER, BM0002 | 1:1000 |
| Fibronectin (FN) | M | Servicebio, GB12091 | 1:1000 |
| Collagen I | R | Servicebio, Gb11022 | 1:1000 |
| FAP | M | Abnova, H00002191-M01 | 1:1000 |
| WDR1 | R | Abcam, ab173574 | 1:1000 |
| ERK1/2 | R | Abclonal, A22447 | 1:1000 |
| P53 | R | Abclonal, A22449 | 1:1000 |
| YAP | R | Cell Signaling Technology, #14074 | 1:1000 |
| CD9 | R | Abclonal, A19027 | 1:1000 |
| CD81 | R | Abclonal, A4863 | 1:1000 |
| CD63 | R | Abclonal, A19023 | 1:1000 |
| HA-tag | M | Proteintech, 66006-2-lg | 1:2000 |
| 3xFLAG-tag | R | Proteintech, 20543-1-ap | 1:2000 |
| GAPDH | M | Proteintech, 60004-1-lg | 1:2000 |
| HRP-Goat anti-R IgG | G | Abclonal, AS014 | 1:5000 |
| HRP-Goat anti-M IgG | G | Abclonal, AS003 | 1:5000 |
|  | | | |
| IF antibody | | | |
| YAP | R | Cell signaling technology, #14074 | 1:200 |
| a-SMA | M | BOSTER, BM0002 | 1:200 |
| Collagen I | R | Servicebio, Gb11022 | 1:200 |
| Alexa Fluor488 Goat anti-R IgG | G | Servicebio, Gb25303 | 1:500 |
| Alexa Fluor488 Goat anti-M IgG | G | Servicebio, Gb25301 | 1:500 |
| Cyanine3 Goat anti-R IgG | G | Servicebio, Gb21303 | 1:500 |
| Cyanine3 Goat anti-M IgG | G | Servicebio, Gb31301 | 1:500 |
|  | | | |
| IHC antibody | | | |
| a-SMA | M | BOSTER, BM0002 | 1:200 |
| Fibronectin (FN) | M | Servicebio, GB12091 | 1:1000 |
| HRP-Goat anti-R IgG | G | Servicebio, G1213-100UL | 1:200 |
| HRP-Goat anti-M IgG | G | Servicebio, G1214-100UL | 1:200 |
|  | | | |
| FACS antibody | | | |
| CD11b | M | BD Horizon, 562950 | 0.06 ug/test |
| Ly6C/Ly6G | M | BD Horizon, 552093 | 0.06 ug/test |
| F4/80 | M | BD Horizon, 565410 | 0.06 ug/test |
| CD16/32 | M | BD Horizon, 553141 | 0.05 ug/test |
| CD45 | M | BD Horizon, 557659 | 0.06 ug/test |

M, Mouse; R, Rabbit; G, Goat

**Table S3.**

Proteins interacted with *WDR1* in p-HSCs identified by mass spectrometry

| **Accession** | **Protein Name** | **Gene** | **log2 intensity pHSCs-WDR1** | **log2 intensity pHSCs-IgG** | **log2 FC** |
| --- | --- | --- | --- | --- | --- |
| O75083 | WDR1_HUMAN | WDR1 | 30.5225298 | 26.7617985 | 3.76073127 |
| P01861 | IGHG4_HUMAN | IGHG4 | 29.1837846 | 20.0102049 | 9.17357966 |
| Q9GZM8 | NDEL1_HUMAN | NDEL1 | 26.737248 | 18.1170546 | 8.62019339 |
| Q96BY7 | ATG2B_HUMAN | ATG2B | 26.1201673 | 19.8750301 | 6.2451372 |
| Q9Y5S2 | MRCKB_HUMAN | CDC42BPB | 27.4151425 | 21.5419852 | 5.87315737 |
| Q96S97 | MYADM_HUMAN | MYADM | 25.2037038 | 19.6141032 | 5.58960055 |
| Q9UGP4 | LIMD1_HUMAN | LIMD1 | 26.4206779 | 20.8412265 | 5.57945145 |
| Q86X02 | CDR2L_HUMAN | CDR2L | 27.2288517 | 21.7156537 | 5.513198 |
| P52732 | KIF11_HUMAN | KIF11 | 24.2323512 | 18.7929737 | 5.43937756 |
| Q96T58 | MINT_HUMAN | SPEN | 24.9553118 | 19.534851 | 5.42046082 |
| P62736 | ACTA_HUMAN | ACTA2 | 24.5304697 | 19.1227916 | 5.40767805 |
| Q9H173 | SIL1_HUMAN | SIL1 | 24.8142605 | 19.5408414 | 5.27341915 |
| P60059 | SC61G_HUMAN | SEC61G | 23.2006316 | 18.2010851 | 4.99954651 |
| Q53SF7 | COBL1_HUMAN | COBLL1 | 24.8186064 | 19.8699274 | 4.948679 |
| Q9UMS6 | SYNP2_HUMAN | SYNPO2 | 24.203293 | 19.2556037 | 4.94768934 |
| Q93052 | LPP_HUMAN | LPP | 31.5357048 | 26.6637021 | 4.87200271 |
| P43005 | EAA3_HUMAN | SLC1A1 | 24.4236781 | 19.6214107 | 4.80226747 |
| Q9H6T3 | RPAP3_HUMAN | RPAP3 | 23.7008114 | 18.9119954 | 4.78881594 |
| Q9P2B4 | CT2NL_HUMAN | CTTNBP2NL | 24.7186353 | 19.9965893 | 4.72204596 |
| A0A024RBG1 | NUD4B_HUMAN | NUDT4B | 23.660251 | 19.1455483 | 4.51470266 |
| P51786 | ZN157_HUMAN | ZNF157 | 23.9568632 | 19.4872233 | 4.4696399 |
| O76071 | CIAO1_HUMAN | CIAO1 | 23.9649035 | 19.6026763 | 4.36222714 |
| Q06190 | P2R3A_HUMAN | PPP2R3A | 25.4788642 | 21.1899066 | 4.28895757 |
| Q8WU90 | ZC3HF_HUMAN | ZC3H15 | 23.7786635 | 19.5301974 | 4.24846612 |
| P01889 | HLAB_HUMAN | HLA-B | 27.3503488 | 23.1491715 | 4.20117729 |
| Q9NVP1 | DDX18_HUMAN | DDX18 | 24.1209978 | 19.9385089 | 4.18248894 |
| P52435 | RPB11_HUMAN | POLR2J | 23.1119552 | 19.0470107 | 4.06494443 |
| O15027 | SC16A_HUMAN | SEC16A | 24.7367578 | 20.7006632 | 4.0360946 |
| P15374 | UCHL3_HUMAN | UCHL3 | 23.8423972 | 19.8066585 | 4.03573869 |
| Q09028 | RBBP4_HUMAN | RBBP4 | 23.6322302 | 19.623901 | 4.00832924 |
| Q13033 | STRN3_HUMAN | STRN3 | 23.8028208 | 19.7953701 | 4.0074507 |
| Q29RF7 | PDS5A_HUMAN | PDS5A | 24.5245637 | 20.534863 | 3.98970075 |
| P19388 | RPAB1_HUMAN | POLR2E | 22.2476274 | 18.2973115 | 3.95031589 |
| Q9Y385 | UB2J1_HUMAN | UBE2J1 | 22.9607034 | 19.0228275 | 3.9378759 |
| P55287 | CAD11_HUMAN | CDH11 | 25.8337628 | 21.9020541 | 3.93170863 |
| P26232 | CTNA2_HUMAN | CTNNA2 | 23.6076103 | 19.6987343 | 3.90887596 |
| Q86W92 | LIPB1_HUMAN | PPFIBP1 | 23.7608093 | 19.8535532 | 3.90725607 |
| Q9BSD7 | NTPCR_HUMAN | NTPCR | 23.2224795 | 19.3483976 | 3.87408184 |
| Q9NRL3 | STRN4_HUMAN | STRN4 | 25.1050558 | 21.245569 | 3.85948688 |
| Q9BQ67 | GRWD1_HUMAN | GRWD1 | 23.4122215 | 19.5611956 | 3.85102582 |
| Q66K74 | MAP1S_HUMAN | MAP1S | 23.1104425 | 19.305502 | 3.8049405 |
| Q96KP1 | EXOC2_HUMAN | EXOC2 | 23.0867673 | 19.2909852 | 3.79578212 |
| Q9P0J7 | KCMF1_HUMAN | KCMF1 | 23.0073921 | 19.2379132 | 3.76947891 |
| Q8N1F7 | NUP93_HUMAN | NUP93 | 23.2325708 | 19.4860792 | 3.7464916 |
| P12273 | PIP_HUMAN | PIP | 22.8233571 | 19.0999083 | 3.72344879 |
| P84095 | RHOG_HUMAN | RHOG | 23.3782218 | 19.707391 | 3.67083078 |
| Q6NXE6 | ARMC6_HUMAN | ARMC6 | 22.8420518 | 19.2161386 | 3.62591322 |
| Q58FF8 | H90B2_HUMAN | HSP90AB2P | 23.6333394 | 20.0181113 | 3.61522812 |
| P29558 | RBMS1_HUMAN | RBMS1 | 23.0690065 | 19.4578074 | 3.61119908 |
| P63162 | RSMN_HUMAN | SNRPN | 22.3014538 | 18.7293772 | 3.57207659 |
| Q9H9J2 | RM44_HUMAN | MRPL44 | 23.3958447 | 19.8308077 | 3.56503703 |
| Q9UBT2 | SAE2_HUMAN | UBA2 | 23.0806165 | 19.5565707 | 3.52404577 |
| P40222 | TXLNA_HUMAN | TXLNA | 22.6871399 | 19.1700148 | 3.51712502 |
| P49773 | HINT1_HUMAN | HINT1 | 23.3114667 | 19.8015513 | 3.50991545 |
| P19387 | RPB3_HUMAN | POLR2C | 22.8427999 | 19.3356993 | 3.50710065 |
| Q92626 | PXDN_HUMAN | PXDN | 23.9465487 | 20.4879735 | 3.4585752 |
| Q9UIL1 | SCOC_HUMAN | SCOC | 22.7674724 | 19.3325596 | 3.43491282 |
| Q96EP0 | RNF31_HUMAN | RNF31 | 23.2381225 | 19.8114847 | 3.42663773 |
| P51116 | FXR2_HUMAN | FXR2 | 22.6079037 | 19.1859623 | 3.42194141 |
| Q96RQ3 | MCCA_HUMAN | MCCC1 | 22.8131548 | 19.4037185 | 3.40943642 |
